# Interpretable machine learning classifies β-lactamase subclasses and prioritizes boundary variants for antimicrobial resistance surveillance

**DOI:** 10.3389/fphar.2026.1876559

**Published:** 2026-09-25

**Authors:** Rahul Kaushik, Suyong Re

**Affiliations:** Laboratory of In-silico Design, Artificial Intelligence Center for Health and Biomedical Research, National Institutes of Biomedical Innovation, Health and Nutrition (NIBN), Osaka, Japan

**Keywords:** antimicrobial resistance, computational pharmacology, drug resistance mechanisms, explainable machine learning, resistance surveillance, β-lactamase

## Abstract

**Introduction:**

Antimicrobial resistance is a major global health threat, and β-lactamase-mediated inactivation of β-lactam antibiotics remains a leading cause of treatment failure. Rapid identification of emerging β-lactamase variants and improved understanding of their functional diversity are therefore essential for resistance surveillance and therapeutic development.

**Methods:**

Here, we developed an interpretable machine learning framework to classify major Class A β-lactamase subclasses (TEM, SHV, and CTX-M) and distinguish them from non-β-lactamase hydrolases using multidimensional protein descriptors. Descriptors based on sequence composition, physicochemical properties, residue accessibility, and structural topology were evaluated to identify a minimal panel of 30 descriptors while preserving biological interpretability.

**Results:**

The selected descriptors provided strong discriminatory power and were linked to steric environment, hydrophobicity, flexibility, solvent exposure, and residue interaction patterns, consistent with roles in substrate recognition and catalysis. Projection into reduced descriptor space identified transitional variants near subclass boundaries, including CTX-M/SHV convergence zones and non-β-lactamase hydrolases with partial β-lactamase-like signatures. Representative case studies indicated that some apparent misclassifications may reflect annotation inconsistencies, scaffold convergence, or early adaptive trajectories, highlighting candidates for experimental follow-up. External validation using 271 independent proteins annotated as non-β-lactamase hydrolases demonstrated 96.95% specificity, with only limited false-positive assignments. Most incorrect classifications showed weak statistical support, indicating that confidence filtering could further improve screening performance.

**Discussion:**

Overall, this study demonstrates that compact descriptor-based models can provide accurate and interpretable classification of β-lactamase-related proteins while identifying emerging boundary variants relevant to antimicrobial resistance surveillance. The framework offers a scalable approach for protein annotation, monitoring resistance evolution, and supporting future inhibitor development.

## 1 Introduction

Antimicrobial resistance (AMR) is now recognized as one of the most serious threats to global health, food security, and sustainable healthcare systems. The accelerated emergence of multidrug-resistant bacterial pathogens has been driven largely by extensive and frequently inappropriate antibiotic exposure in human medicine, veterinary practice, agriculture, and environmental reservoirs. Consequently, infections that were once readily treatable increasingly require prolonged hospitalization, combination therapy, or toxic last-line agents, resulting in higher mortality, greater healthcare expenditure, and substantial socioeconomic burden. Recent estimates indicate that bacterial AMR was directly associated with more than one million deaths globally in 2019, while future projections suggest that resistant infections may account for up to 10 million deaths annually by 2050 if effective countermeasures are not implemented (de Kraker et al., 2016; Murray et al., 2022; Naghavi et al., 2024; Kant et al., 2024). These trends underscore the urgent need not only to catalogue resistance determinants, but to understand their molecular behavior in ways that can guide therapeutic innovation.

Among the major mechanisms of bacterial resistance, enzymatic inactivation of β-lactam antibiotics by β-lactamases remains one of the most clinically consequential. β-Lactam agents, including penicillins, cephalosporins, monobactams, and carbapenems, constitute a central pillar of antibacterial therapy because of their broad utility, favorable pharmacokinetics, and well-established safety profiles. Their widespread use, however, has created strong selective pressure for bacterial enzymes capable of hydrolyzing the β-lactam ring, thereby abolishing antibacterial activity. β-Lactamases are now distributed across diverse Gram-negative and Gram-positive pathogens and have become key drivers of hospital- and community-acquired resistance (Quan et al., 2024; Belay et al., 2024; Oliver et al., 2025; Bush and Bradford, 2020).

Particularly important within this landscape are Ambler Class A serine β-lactamases, a highly adaptable enzyme family that includes TEM, SHV, CTX-M, KPC, SME, GES, and related lineages. Historically, TEM and SHV variants were among the earliest plasmid-mediated β-lactamases to disseminate widely, while CTX-M enzymes subsequently emerged as the dominant global extended-spectrum β-lactamases (ESBLs), reshaping resistance epidemiology in *Enterobacterales*. Many members of these families have evolved expanded hydrolytic activity toward oxyimino-cephalosporins, and some Class A enzymes now exhibit carbapenemase activity, severely constraining treatment options (Bush, 2018; Doi et al., 2017; Lepe and Martínez-Martínez, 2022; Yu et al., 2024). Because β-lactam/β-lactamase inhibitor combinations are increasingly relied upon for therapy, continued diversification of Class A enzymes also threatens the durability of inhibitor-based rescue strategies (Tehrani et al., 2018; Rajer et al., 2022).

Despite decades of biochemical and structural investigation, predicting the functional behavior of newly observed β-lactamase variants remains challenging. Conventional classification systems based on sequence identity, phylogeny, or curated annotation provide valuable lineage information, yet they do not always resolve subtle changes in substrate spectrum, catalytic efficiency, inhibitor susceptibility, or mutational potential. Single amino-acid substitutions can alter active-site electrostatics, loop mobility, solvent accessibility, or residue packing in ways not obvious from sequence comparison alone. Conversely, distantly related proteins may converge upon similar catalytic microenvironments and resistance phenotypes despite limited sequence similarity (Gherardini et al., 2007; Kaushik et al., 2023; Kaushik et al., 2022; Keshri et al., 2019). This creates a practical bottleneck for surveillance programs, genome annotation pipelines, and computational drug discovery efforts seeking to prioritize high-risk variants.

The pharmacological implications of this limitation are substantial. Modern antibacterial development increasingly depends on accurate anticipation of resistance trajectories so that novel β-lactams, inhibitor scaffolds, adjuvants, or combination regimens can be optimized before widespread clinical failure occurs. Structural biology and molecular simulation have improved understanding of catalytic serine acylation, de-acylation, Ω-loop dynamics, and inhibitor engagement, but these approaches are not always scalable for large sequence datasets generated by genomic surveillance (Tooke et al., 2023; Sherry et al., 2025). There is therefore strong demand for computational frameworks that can rapidly screen protein diversity while preserving mechanistic interpretability relevant to medicinal chemistry and translational pharmacology (D’Arrigo et al., 2024; Sadybekov and Katritch, 2023).

Recent advances in machine learning and artificial intelligence-based approaches offer a powerful route to this objective. When coupled with biologically meaningful molecular descriptors, machine-learning models can integrate sequence-derived, structural, and network-level information into predictive representations of enzyme function (Paton et al., 2025; Song et al., 2024; Abramson et al., 2024; Kaushik et al., 2018; Kaushik and Zhang, 2022a). However, many published models prioritize classification accuracy alone and provide limited explanation of which molecular properties drive predictions. In resistance pharmacology, black-box performance is insufficient: actionable models should indicate why a variant resembles a known resistance class, which descriptors control that resemblance, and how these features relate to substrate access, catalytic geometry, or inhibitor vulnerability. Interpretable learning frameworks such as SHAP-based feature attribution now make such analyses increasingly feasible (Dhibar et al., 2025; Kaushik and Zhang, 2020; Melo et al., 2021; Kaushik and Zhang, 2022b).

Despite the growing application of these computational methods to resistome data, existing models for antimicrobial resistance gene (ARG) annotation generally prioritize large-scale input feature arrays over mechanistic interpretability. Current state-of-the-art architectures can be broadly categorized into deep learning models such as DeepARG (Arango-Argoty et al., 2018), sequence alignment features based/profile Hidden Markov Model (HMM) based classifiers such as AMRFinder+ (Feldgarden et al., 2022), and protein language models (pLMs) leveraging pre-trained transformer embeddings such as PLM-ARG (Wu et al., 2023). While profile HMMs offer localized motif interpretability by mapping alignment conservation weights, deep learning architectures and computationally intensive pLM representations often operate as functional black boxes or sequence-dependent compositional statistics. This variability creates a distinct trade-off between open-ended target discovery and fine-grained intra-family subclass discrimination. Consequently, there remains a critical gap in the literature for lightweight, alignment-free frameworks that can perform precise subclass sorting while preserving manual biochemical interpretability.

In this study, we present an interpretable multidimensional descriptor framework for the characterization of Class A β-lactamases and related bacterial hydrolases. By integrating sequence-composition features, physicochemical autocorrelation terms, residue accessibility metrics, and topology-informed descriptors, we generate a unified representation that captures both global scaffold organization and localized catalytic environments. Through systematic feature reduction and explainable machine learning, we identify a minimal 30-descriptor panel sufficient to discriminate major Class A β-lactamase subclasses from non-β-lactamase hydrolases (represented as “NON” throughout the manuscript) while retaining biological interpretability. Beyond classification, the framework reveals (i) transitional proteins occupying boundary regions between subclasses, (ii) identifies potential annotation inconsistencies, and (iii) detects noncanonical enzymes with partial β-lactamase-like signatures. External validation on taxonomically diverse independent proteins further tests model specificity under realistic screening conditions. These capabilities are directly relevant to antimicrobial resistance surveillance, where early recognition of emerging or misannotated β-lactamase variants can inform genomic monitoring, and laboratory prioritization. By linking descriptor-level signatures to known resistance mechanisms, this work also provides a translational basis for inhibitor design and next-generation antibacterial development. The identified descriptors associated with steric environment, electrostatics, flexibility, and solvent exposure may help in rationalizing role of certain scaffolds in broadening substrate range or evading inhibition. More broadly, the study illustrates how explainable artificial intelligence can complement molecular pharmacology to accelerate discovery strategies against resistant bacterial pathogens.

## 2 Materials and methods

### 2.1 Dataset construction and curation

A curated dataset of Class A β-lactamase protein sequences was constructed to ensure structural integrity, accurate subclass assignment, and representation of clinically relevant diversity. Emphasis was placed on maintaining sequences corresponding to full-length, catalytically competent enzymes within the characteristic molecular range (∼28–35 kDa), thereby ensuring relevance to functional and pharmacological analyses. Publicly accessible and version-controlled repositories were used to ensure reproducibility and traceability.

#### 2.1.1 Sequence retrieval, filtering and refinement

Protein sequences corresponding to TEM, SHV, and CTX-M β-lactamases were retrieved from the UniProt Knowledgebase, including both manually curated (Swiss-Prot) and computationally annotated (TrEMBL) entries (Bateman et al., 2025). Retrieval was restricted to members of the order *Enterobacterales*, encompassing clinically significant hosts associated with β-lactam resistance. Subclass-specific queries were constructed using gene annotations (blaTEM, blaSHV, blaCTX-M), protein names, and enzyme classification (EC 3.5.2.6). The initial dataset comprised 1,184 sequences spanning TEM, SHV, and CTX-M families. To ensure functional relevance, all entries were evaluated for domain integrity using Pfam and InterPro, retaining only sequences containing canonical Class A β-lactamase domains (e.g., PF13354, PF00144, IPR012338). Sequences lacking complete catalytic domain coverage, exhibiting truncations, or showing ambiguous annotation were excluded. Further filtering ensured the absence of non-standard amino acids and retained only sequences with molecular weights consistent with functional β-lactamases. After physicochemical validation and manual curation based quality control 486 sequences met all inclusion criteria. The curated dataset consisted of 213 CTX-M, 171 TEM, and 102 SHV entries, consistent with the contemporary epidemiological dominance of CTX-M enzymes among *Enterobacterales*. Protein existence evidence in UniProt indicated that only four entries had direct protein-level evidence, whereas 482 were supported by homology inference. This imbalance reflects the scale of genome-derived annotation pipelines and emphasizes the need for computational frameworks capable of handling large sequence repositories with limited experimental characterization.

To minimize overrepresentation of highly similar variants while preserving subclass diversity, redundancy reduction was performed using CD-HIT (Li and Godzik, 2006). Because single amino acid substitutions in Class A β-lactamases can substantially alter hydrolytic spectrum, inhibitor susceptibility, catalytic efficiency, or expression behavior, redundancy reduction was intentionally performed at 100% sequence identity rather than conventional lower thresholds. This approach removed exact duplicates while preserving all unique mutational variants. The final non-redundant dataset comprised 274 representative proteins, including 73 TEM, 70 SHV and 131 CTX-M variants. Clustering was conducted independently within each subclass to avoid artificial merging across distinct β-lactamase families, thereby maintaining biologically meaningful subclass distinctions. This dataset captures representative diversity while preserving the structural and functional characteristics relevant to resistance mechanisms. A species-level taxonomic distribution of the curated non-redundant Class A β-lactamase dataset is provided in Supplementary Table S1 and a summary of a stepwise compilation and refinement of Class A β-lactamase sequences from initial retrieval in UniProt to the final curated non-redundant dataset is provided in Table 1.

**TABLE 1:** Stepwise compilation and refinement of class A β-lactamase sequences from initial retrieval in UniProt to the final curated non-redundant dataset.

Processing step	TEM	SHV	CTX-M	Total
Initial retrieval from UniProt	367	240	577	1,184
After subclass classification and preliminary filtering*	171	102	213	486
After 100% identity clustering (CD-HIT clusters)	73	70	131	274
Number of singleton clusters	62	61	107	230
Largest cluster size	69	12	14	—
Mean cluster size (excluding singletons)	9.91	4.56	4.42	—
Final curated non-redundant representatives	73	70	131	274

* Preliminary filtering included domain validation (class A β-lactamase architecture), sequence length threshold (≥200 amino acids), molecular weight 28–35 kDa, removal of sequences containing non-standard residues, and consistent subclass annotation.

#### 2.1.2 Subclass non-redundancy threshold and functional validation

Subclass assignments and final target validation were performed by integrating database metadata curation, strict active-site motif screening, and intra-class sequence identity redundancy thresholds. To eliminate sequence duplicates while preserving every unique point mutation, redundancy reduction was executed independently within each subclass using a strict sequence identity threshold of exactly 100%. This precise intra-class filter compressed the validated cohort of 486 primary sequences down to 274 unique mutational targets, comprising 73 TEM, 70 SHV, and 131 CTX-M variants. Specifically, this non-redundant landscape preserved 62, 61, and 107 single-sequence singleton clusters for TEM, SHV, and CTX-M, respectively, alongside single chosen representatives from the remaining non-singleton duplicate groups. To ensure downstream structural and functional relevance, these 274 cluster-representative variants were simultaneously checked against established database annotations and screened for the absolute preservation of key catalytic motifs. Functional status was verified by tracking the precise spatial conservation of the four-core Ambler Class A β-lactamase catalytic microenvironments: the primary nucleophilic center (^70^STXK), the structural stabilization loop (^130^SDN), the mobile substrate-binding (Ω-loop (^166^EXXXN), and the terminal anchor strand (^234^KTG) (Belay et al., 2024; Lepe and Martínez-Martínez, 2022; Radojković and Ubbink, 2024). Any sequence failing to meet this 100% motif preservation rule or exhibiting structural truncations was excluded. This multi-layered validation guaranteed that each entry in the final 274-variant dataset represents a functionally competent enzyme with reliable subclass identity, supporting downstream biophysical descriptor calculation.

#### 2.1.3 Compilation and architectural composition of the hard-negative control dataset

To ensure that the multi-dimensional descriptor signatures capture precise, lineage-specific resistance mechanisms rather than generic bacterial enzyme architecture, a matched structural control cohort of 274 non-target bacterial proteins was assembled (designated uniformly as the “NON” dataset). Rather than utilizing distant or superficial sequence negatives that can artificially inflate machine learning metrics, this control matrix was explicitly populated with highly informative hard negatives and functional decoys (Supplementary Table S2). The dataset was curated from reviewed UniProt entries and structured across key biophysical categories to present a stringent screening boundary for the classifier. These categories include:

##### 2.1.3.1 Cell-wall peptidases and penicillin-binding proteins (PBPs; 13.87%)

Incorporating true zinc- and serine-dependent DD-peptidases, carboxypeptidases, and endopeptidases (e.g., P00733, P0C0T5, P39042). Because PBPs are the direct evolutionary ancestors of β-lactamases and share highly conserved penicillin-binding cleft geometries, their inclusion forces the 30 biophysical descriptors to learn true resistance-level subclass traits rather than generic cell-wall processing frameworks.

##### 2.1.3.2 Classical serine hydrolases, esterases, and lipases (42.70%)

Featuring functional serine-nucleophile cascades such as trypsin, streptogrisin-A, lysyl endopeptidase, and carboxylesterases (e.g., BioH, TesA). These entries validate the model’s capacity to differentiate generalized macromolecular cleavage tunnels from the rigid pocket geometries required to coordinate a cyclic amide ring.

##### 2.1.3.3 Lactonases, deacetylases, and non-target β-lactamases (25.18%)

Incorporating cyclic ester-cleaving lactonases, glyoxalase II enzymes (GloB), and active, non-target β-lactamases spanning alternative Ambler classes (such as Class B MBLs VIM-1/NDM-1, Class D oxacillinases OXA-48/OXA-23, and Class A carbapenemases KPC-2/GES-5). The inclusion of alternative Class A carbapenemases serves as an ultimate specificity filter, ensuring the pipeline executes strict intra-family subclass discrimination.

##### 2.1.3.4 Phosphatases and nucleases (18.25%)

Including diverse functional decoys (e.g., PtpB, SuhB, Exo III) to benchmark model thresholding against non-specific background hydrolase properties.

To maintain absolute descriptor reliability, only high-quality structural profiles were included. The operational inclusion criteria required: (i) the total absence of non-standard amino acid residues that could distort spatial or biochemical calculations, (ii) fully continuous polypeptide backbones lacking structural breaks that might confound graph-theoretic or residue-contact topology metrics, and (iii) sequence dereplication at an 80% identity threshold using CD-HIT to reduce redundancy and guarantee taxonomic diversity. Experimentally determined structures from the Protein Data Bank (PDB) were prioritized for direct structural representation. Where required to preserve dataset completeness and balance, proteins lacking suitable resolved crystal coordinates were modeled using the standalone AlphaFold3 platform under the same confidence criteria (mean pLDDT ≥90), total fold continuity, and structural integrity) applied to the target β-lactamase variants. This highly balanced and mechanistically challenging control set establishes a robust statistical safety net, ensuring the downstream framework remains highly conservative when challenged with unfamiliar bacterial hydrolase backgrounds.

### 2.2 Structural characterization of class A β-lactamases and hybrid model assembly

To complement sequence-level curation and ensure structural interpretability, a unified three-dimensional structural framework was established across the entire curated Class A β-lactamase cohort, the non-β-lactamase control dataset (NON), and the independent external validation set. Because antibiotic resistance phenotypes are heavily governed by the spatial organization of catalytic residues, active-site cavity geometry, substrate access pathways, and loop configurations, incorporating consistent 3D coordinate metadata is essential. This characterization pipeline prioritized experimentally resolved structures wherever available and deployed high-confidence computational models where experimental data were absent.

#### 2.2.1 Identification of experimentally determined structures and AlphaFold3 structural modeling

All 274 non-redundant TEM, SHV, and CTX-M variants were systematically screened against structures deposited in the Protein Data Bank (PDB) (Vallat et al., 2026) using DIAMOND (Buchfink et al., 2015) to identify closest structural homologs. Sequences demonstrating more than 95% sequence identity across full-length alignment coverage to the experimental structure(s) were considered to possess reliable templates suitable for direct structural representation. To account for variable numbers of flexible N- and C-terminal residues that are frequently absent in crystal structures compared to full full-length query sequences, this 95% identity cutoff was selected to maximize the inclusion of relevant experimental structures. Importantly, to ensure this alignment tolerance did not introduce variance into the functional active site, a strict 100% identity match was simultaneously required across all core catalytic microenvironments ^70^STXK, ^130^SDN, the Ω-loop, and ^234^KTG. Any template exhibiting sequence variation or missing coordinates within these catalytic motifs was discarded in favor of exact structural modeling via AlphaFold3. Following this sequence similarity screening, high-confidence experimental structural templates were successfully assigned to 220 out of the 274 β-lactamase variants (80.3%). The precise subclass breakdown of these experimental assignments comprised: TEM Subclass: 64 variants mapped closely to canonical TEM crystal structures. SHV Subclass: 61 variants aligned predominantly with established SHV structural representatives. CTX-M Subclass: 95 variants demonstrating extensive correspondence with resolved CTX-M enzyme templates. The remaining 54 variants (19.7%), which fell below the identity/full-coverage threshold due to heavier sequence diversification, partial alignments, or recent evolutionary mutations under cephalosporin selection pressure, were modeled using the standalone AlphaFold3 (AF3) platform. Variants requiring computational modeling were more frequent within the CTX-M subclass, reflecting its rapid adaptive expansion. Predicted structures were evaluated using established local distance difference test (pLDDT) scores. Only models demonstrating high structural confidence (mean pLDDT ≥ 90), total fold continuity, and exact preservation of the canonical Class A β-lactamase fold architecture were retained.

#### 2.2.2 Structural superimposition, active-site analysis, and divergence quantification

All experimental and computationally modeled structures were subjected to systematic multi-scale structural analysis. Secondary structure elements were assigned via DSSP (Hekkelman et al., 2025). To quantify fold conservation and identify subclass-specific geometric dispersion, global structural superimpositions were performed using TM-align (Zhang and Skolnick, 2005) against three subclass prototype reference scaffolds: TEM-1 (PDB: 1ZG4), SHV-1 (PDB: 4JPM), and CTX-M-15 (PDB: 4HBT). These structural overlays revealed that the TEM subclass displayed the tightest structural conservation, followed closely by SHV, whereas the CTX-M variants exhibited the highest degree of conformational dispersion and localized structural plasticity. During superimposition, particular emphasis was placed on the catalytic microenvironment. Key core residues required for β-lactam coordination, acylation, and diacylation, specifically Ser70, Lys73, Ser130, Glu166, and Asn170 (Ambler numbering), were mapped across all 274 structural scaffolds to verify the absolute spatial preservation of catalytic geometry. Visual inspections and structural validation were conducted using ChimeraX (Meng et al., 2023), supplemented by automated feature-extraction pipelines to compute downstream multi-dimensional descriptors.

### 2.3 Sequence-derived biochemical characterization of class A β-lactamases

To systematically characterize the intrinsic biochemical properties of the curated Class A β-lactamases, sequence-derived descriptors were used to capture multiple layers of molecular information embedded within the primary structure, as summarized in Table 2. Rather than serving as abstract numerical values, these descriptors represent quantifiable proxies for physicochemical behavior, residue organization, and spatial distribution patterns that are directly linked to protein stability, substrate interaction, and catalytic efficiency. This approach enables the translation of sequence information into biologically interpretable descriptors relevant to antibiotic recognition and hydrolysis. A comprehensive descriptor space was constructed for all 274 sequences, integrating multiple complementary categories that collectively describe global composition, physicochemical tendencies, and sequence-order-dependent effects. This multi-scale representation allows interrogation of both broad evolutionary constraints and localized sequence descriptors that contribute to functional specialization among β-lactamase subclasses.

**TABLE 2:** Mechanistically annotated summary of sequence- and structure-derived descriptors used for characterization of class A β-lactamases. Each descriptor category is mapped to its functional relevance in enzyme stability, substrate recognition, catalytic activity, and inhibitor interaction.

Category	Descriptor type	Mechanistic relevance	Count
Global composition	Amino acid composition (AAC)	Residue-level biases influencing charge distribution, catalytic residue availability	20
	Dipeptide composition (DPC)	Local residue pairing linked to backbone stability and active-site proximal interactions	400
	Tripeptide composition (TPC)	Short motifs capturing conserved catalytic signatures and substrate recognition patterns	8,000
Grouped composition	Grouped AAC (GAAC)	Residue polarity, charge, hydrophobicity governing stability and ligand interaction	9
Physico-chemical	MW, pI, GRAVY, instability index	Electrostatics, solubility, hydrophobic balance, and overall structural stability	4
CTD descriptors	Composition (C)	Bulk physicochemical environment and fold-level biochemical identity	21
	Transition (T)	Sequence heterogeneity and transitions between physicochemical states	21
	Distribution (D)	Spatial positioning of key residue classes linked to catalytic regions	105
Autocorrelation	Moreau–Broto	Long-range propagation of physicochemical properties affecting fold stability	240
	Moran	Global spatial correlation reflecting coordinated structural organization	240
	Geary	Local physicochemical fluctuations capturing microenvironment variability	240
Structural geometry	SASA, Avg. SASA, radius of gyration	Solvent exposure and compactness governing substrate accessibility	3
Secondary structure	Helix, sheet, coil (%)	Scaffold rigidity and active-site flexibility	3
Topology (RCN)	Degree, clustering, path length, betweenness	Internal connectivity and communication affecting catalytic efficiency	4
Active site	Volume, druggability, hydrophobicity, residue composition	Catalytic cavity properties governing substrate accommodation and inhibitor binding	4
Total	—	Integrated multi-scale descriptor space	9,314

#### 2.3.1 Global compositional profiles

Global compositional descriptors quantify the overall amino acid makeup of each protein, providing a first-order approximation of its biochemical identity. These descriptors capture residue-level biases that influence folding propensity, electrostatic environment, and interaction potential with β-lactam substrates. Amino acid composition reflects the relative abundance of individual residues (Emonts and Buyel, 2023; Kaushik and Re, 2026), while dipeptide and tripeptide compositions extend this representation to local sequence patterns (Das Gupta et al., 2016; Kaushik et al., 2024). These higher-order motifs are particularly relevant for identifying conserved signatures associated with catalytic function, including sequence fragments proximal to the active-site architecture. Such compositional patterns provide insight into how residue preferences shape both structural scaffolding and functional specificity across subclasses.

#### 2.3.2 Global physicochemical properties

To contextualize sequence composition in terms of biochemical behavior, global physicochemical parameters were evaluated. These descriptors summarize how each protein is expected to behave under physiological conditions, including properties that influence folding stability, solubility, and interaction with charged or hydrophobic substrates. Key properties include molecular weight and isoelectric point, which define overall size and charge characteristics, as well as indices related to structural stability and hydrophobicity (Qing et al., 2022; Milchevskiy et al., 2024). For instance, hydropathy-based measures reflect the balance between hydrophobic and hydrophilic residues, which is critical for maintaining the integrity of the catalytic pocket and mediating substrate access. Similarly, stability-related indices provide indirect insight into the robustness of the enzyme scaffold under varying environmental conditions.

#### 2.3.3 Composition-transition-distribution (CTD) descriptors

To capture how physicochemical properties are organized along the sequence, Composition–Transition–Distribution (CTD) descriptors were employed. These descriptors extend beyond global averages by quantifying the arrangement and positional distribution of residues grouped by shared properties such as polarity, hydrophobicity, and volume (Kaushik and Zhang, 2020; Elreify et al., 2025; Mall et al., 2025). This framework enables assessment of how specific residue classes are distributed across the sequence, how frequent the transitions occur between different property groups, and where key residues are localized. Such information is particularly relevant for understanding the spatial organization of regions contributing to substrate binding, loop flexibility, and catalytic site accessibility.

#### 2.3.4 Sequence-order and spatial correlation descriptors

While compositional descriptors capture residue frequency, they do not account for long-range interactions within the sequence. To address this, autocorrelation-based descriptors were used to quantify how physicochemical properties are distributed across defined sequence intervals. These descriptors measure correlations between residues separated by varying distances, providing insight into periodicity, clustering, and long-range dependencies of biochemical properties. Such patterns are indicative of structural organization and can reflect the coordinated arrangement of residues that stabilize the protein fold or contribute to the formation of the catalytic environment. By incorporating multiple autocorrelation formulations, both global trends and localized variations in physicochemical properties are captured. This enables a more nuanced representation of how sequence-level descriptors translate into structural and functional behavior (Elreify et al., 2025; Zhou et al., 2025).

Collectively, the sequence-derived descriptors provide a high-resolution biochemical representation of Class A β-lactamases, encompassing compositional biases, physicochemical behavior, and spatial organization of residues. This integrated descriptor space forms the basis for subsequent analyses aimed at identifying the molecular determinants of subclass differentiation and their relationship to antibiotic resistance mechanisms.

### 2.4 Structure-derived biophysical characterization of class A β-lactamases

To complement sequence-derived biochemical and structural descriptors were incorporated to capture the three-dimensional organization of Class A β-lactamases. Given that antibiotic hydrolysis is governed by the spatial arrangement of catalytic residues, substrate accessibility, and the physicochemical environment of the active site, structural characterization provides a direct link between molecular architecture and resistance phenotype. A unified set of descriptors was extracted from both experimentally determined and high-confidence modeled structures, enabling consistent analysis across all variants. These descriptors span multiple scales, from global fold architecture and surface exposure to internal connectivity and catalytic pocket properties, thereby providing a comprehensive representation of the structural determinants underlying enzyme function.

#### 2.4.1 Global fold architecture and surface properties

Global structural descriptors were used to quantify overall fold organization and solvent interaction. Secondary structure composition was determined to assess the relative contribution of α-helices, β-sheets, and loop regions, which collectively define the conserved α/β hydrolase scaffold characteristic of Class A β-lactamases. To guarantee workflow reproducibility across all curated target, control, and independent external validation datasets, these calculations were executed by running the standalone STRIDE (Secondary Structure Identification) binary engine via a programmatic Python subprocess. The STRIDE output files were parsed to extract standard 3-state secondary structure ratios (Helix_Pct, Sheet_Pct, and Coil_Pct).

Variations in loop content are particularly relevant, as these regions often contribute to substrate recognition and active-site flexibility. Surface exposure was evaluated through solvent-accessible surface area (Total_SASA, Avg_SASA), derived from the parsed STRIDE atomic exposure lines, providing insight into protein–solvent interactions and potential substrate access pathways. In parallel, structural compactness was assessed through the radius of gyration. This metric was computed directly from coordinate text vectors using an automated arithmetic script within the Bio.PDB module of BioPython (v1.81), calculating the exact root-mean-square distance of all Cα carbon atoms from the protein’s geometric center of mass. Together, these descriptors capture how global structural organization influences enzyme stability and accessibility of the catalytic site.

#### 2.4.2 Internal connectivity and structural topology

To evaluate the internal organization of the protein fold, structures were analyzed as residue-level interaction networks. In this representation, residues form interconnected networks based on spatial proximity, enabling quantification of structural cohesion and communication pathways within the protein. To construct these spatial topologies, pairwise Euclidean distances between all intra-chain Cα coordinates were calculated using the distance-matrix utilities in the scipy.spatial.distance package. An unweighted graph edge was constructed between two residue nodes if their spatial distance fell within a standard structural cutoff threshold of 8.0 Å (strictly excluding self-loops). The resulting adjacency matrices were mapped and analyzed using the NetworkX graph library (v3.1) to compute four core topological descriptors: (i) Average Degree (Avg_Degree): Measures localized packing density and coordinate isolation; (ii) Average Clustering Coefficient (Clustering_Coeff): Quantifies tight interconnections of a residue’s local spatial neighbors; (iii) Average Shortest Path Length (Avg_Path_Length): Reflects overall network efficiency and fold compactness. For any structurally fragmented models, path length calculations were automatically restricted to the largest connected component subgraph; and (iv) Mean Betweenness Centrality (Mean_Betweenness): Quantifies intramolecular communication efficiency, identifying backbone communication hubs flanking active-site loops.

These network-based descriptors are particularly relevant for understanding how structural perturbations propagate across the protein, enabling identification of structural regions that act as stabilizing cores or flexible communication hubs for maintaining catalytic competence and adapting to substrate variability.

#### 2.4.3 Catalytic pocket architecture and functional microenvironment

Given the central role of the active site in β-lactam hydrolysis, focused characterization of catalytic pockets was performed to quantify descriptors directly linked to substrate binding and inhibitor interaction. Local cavity profiling, pocket isolation, and microenvironmental calculations were executed using the specialized fpocket (v4.0) alpha-sphere pocket detection algorithm. To isolate the genuine, functional active-site pocket from superficial surface clefts, a customized coordinate filtration module was implemented within the python script. The pipeline verified the exact spatial presence of the invariant nucleophilic residue Ser70 (Ambler numbering). Once the unique active-site cavity was confirmed, its localized properties were extracted directly from the baseline output text matrices:

1. Active Site Volume (AS_Volume): Reflects the geometric steric capacity to accommodate substrates of varying size, calculated using Monte Carlo sampling inside the pocket coordinates.
2. Active Site Hydrophobicity (AS_Hydrophobicity): Represents the local chemical pocket environment calculated using specialized hydrophobicity score vectors.
3. Active Site Druggability (AS_Druggability): Estimates the overall potential for high-affinity ligand binding, providing a pharmacologically relevant measure of inhibitor accessibility.

These descriptors collectively capture the geometric and physicochemical properties of the catalytic microenvironment, enabling comparison of how different subclasses accommodate substrates and contribute to expanded-spectrum resistance.

Mechanistically annotated summaries of sequence- and structure-derived descriptors used to characterize Class A β-lactamases are provided in Table 2. Each descriptor category is mapped to its functional relevance in enzyme stability, substrate recognition, catalytic activity, and inhibitor interaction. The integration of global structural descriptors, internal connectivity metrics, and active-site descriptors, provides a multi-scale representation of β-lactamase architecture. This framework enables a direct linkage between structural organization and catalytic function, supporting mechanistic interpretation of resistance phenotypes across TEM, SHV, and CTX-M subclasses.

### 2.5 Evaluation of protein language model–derived latent representations

To assess whether deep sequence- and structure-derived latent representations provide additional discriminatory power beyond classical biophysical descriptors, we evaluated embeddings generated from state-of-the-art protein language models (pLMs). This analysis was designed as a comparative benchmarking step to determine the extent to which high-dimensional learned representations capture subclass-specific signatures relevant to β-lactamase-mediated antibiotic resistance.

#### 2.5.1 Sequence-based latent representations (ProtT5)

Sequence embeddings were generated using the ProtT5-XL-UniRef50 model, a transformer-based architecture trained on large-scale protein sequence databases (Elnaggar, 2021). Protein sequences were tokenized at the residue level, and contextual embeddings were extracted from the final encoder layer, yielding residue-level representations of dimension 1,024. To obtain a fixed-length representation for each protein, mean pooling was applied across the sequence length (excluding padding tokens), resulting in a global embedding vector capturing evolutionary and contextual sequence signatures.

#### 2.5.2 Structure-aware multimodal representations (ESM-3)

To incorporate structural context, embeddings were generated using ESM-3 (esm3_sm_open_v1), a multimodal model capable of jointly processing sequence and backbone coordinate information (Hayes et al., 2025). Protein structures were formatted to include atomic coordinates (N, Cα, C, O) alongside sequence inputs. Latent representations (1,536 dimensions) were extracted using the model’s embedding configuration, followed by mean pooling across residues to generate a unified structural-functional representation for each protein.

#### 2.5.3 Construction of integrated latent descriptor space

The ProtT5 and ESM-3 embeddings were concatenated to form a unified 2,560-dimensional latent descriptor space. All descriptors were standardized using Z-score normalization to ensure comparability across modalities. This integrated representation was intended to capture complementary evolutionary and structural signals within a single descriptor space.

#### 2.5.4 Exploratory analysis of latent descriptor space

The high-dimensional latent space was subjected to exploration analysis to evaluate its information density, redundancy, and subclass separability. Variance-based filtering was applied to identify low-information dimensions, followed by correlation analysis to quantify redundancy within and between embedding modalities. To assess intrinsic clustering behavior, a non-linear dimensionality reduction technique, Uniform Manifold Approximation and Projection (UMAP) (Healy and McInnes, 2024), was employed to visualize the organization of TEM, SHV, and CTX-M variants in the latent space. These analyses were used to determine whether pLM-derived representations inherently capture subclass-specific structural and functional differentiation.

#### 2.5.5 Role of latent representations in the final framework

The pLM-derived embeddings were evaluated alongside classical biochemical and structural descriptors to determine their contribution to subclass discrimination. As detailed in Section 3.4, the latent representations exhibited substantial redundancy and did not improve classification performance beyond that achieved using the reduced, interpretable descriptor set. Accordingly, these embeddings were not incorporated into the final predictive framework, and subsequent analyses focus on the mechanistically interpretable descriptor space.

### 2.6 Descriptor refinement and mechanistic attribution framework

To derive a biologically meaningful and non-redundant representation of β-lactamase functional diversity, a two-stage framework combining dimensionality reduction and descriptor attribution was implemented on the integrated dataset comprising Class A β-lactamases and bacterial hydrolases.

#### 2.6.1 Descriptor space refinement and statistical selection

The initial high-dimensional descriptor space was first standardized and subjected to Principal Component Analysis (PCA) to calculate the orthogonal axes of biophysical variance. The cumulative variances captured at 50%, 75%, and 90% thresholds were examined to characterize the intrinsic dimensionality of the dataset and to delineate the hierarchy of structural and physicochemical determinants governing enzyme diversity. For each principal component, descriptors with the highest absolute loading scores were extracted as primary contributors to variance. To ensure robustness and eliminate redundancy, these descriptors were filtered for uniqueness and further screened using pairwise correlation analysis. Subsequently, statistical validation was performed using analysis of variance (ANOVA) to confirm that the retained descriptors exhibit significant differences (p < 0.05) across TEM, SHV, CTX-M, and non-β-lactamase hydrolase (NON) groups. This process resulted in a reduced set of high-impact descriptors representing the core biochemical and structural descriptors underlying subclass differentiation.

#### 2.6.2 Descriptor attribution and identification of minimal functional signatures

To quantify the contribution of individual descriptors to subclass discrimination, an interpretability-driven framework based on SHapley Additive exPlanations (SHAP) was employed (Lundberg et al., 2020). A Random Forest classifier was trained on the refined descriptor set to establish a stable predictive model, enabling decomposition of descriptor contributions within a non-linear decision space. Shapley values were computed using the TreeExplainer algorithm, providing a sample-wise and class-specific estimate of descriptor importance while accounting for descriptor interactions. Global descriptor importance was determined from the mean absolute Shapley values across all proteins, while subclass-specific contributions were evaluated to identify distinct biophysical signatures associated with TEM, SHV, CTX-M, and NON (non-β-lactamase hydrolases). This analysis enabled the extraction of a minimal and interpretable subset of descriptors that collectively define the functional and structural identity of Class A β-lactamases. Importantly, these descriptors represent mechanistically relevant descriptors, including active-site accessibility, electrostatic distribution, hydrophobic packing, and structural connectivity, which are directly linked to substrate specificity and catalytic efficiency.

#### 2.6.3 Integration with structural and functional interpretation

While descriptor attribution provides a quantitative measure of descriptor importance, it does not inherently capture the global organization of the enzyme landscape. Therefore, the SHAP-identified descriptors were further evaluated using dimensionality reduction and clustering approaches (Section 2.8) to assess whether the derived biophysical signatures translate into distinct and separable functional groups. This integrated framework ensures that descriptor selection is not only statistically robust but also mechanistically interpretable, thereby bridging the gap between high-dimensional data representation and pharmacologically relevant insights into β-lactamase-mediated antibiotic resistance.

### 2.7 Descriptor validation, classification, and consistency analysis

To evaluate the robustness, interpretability, and translational relevance of the SHAP-identified biophysical descriptors, an integrated validation framework was implemented combining dimensionality reduction and predictive modeling. This approach was designed to assess whether descriptor-derived signatures consistently capture subclass-specific functional differentiation and align with, or extend beyond, sequence-based evolutionary relationships.

#### 2.7.1 Multidimensional projection and cluster validation

The selected descriptor space was projected into lower-dimensional representations to assess intrinsic separability of TEM, SHV, CTX-M, and NON groups. Principal Component Analysis (PCA) was applied to evaluate global variance structure and determine whether subclass differentiation can be resolved through linear combinations of biochemical and structural descriptors. UMAP was additionally utilized to preserve both local and global topology, providing a stable visualization of subclass clustering and identification of transitional variants. Together, these approaches establish whether the reduced descriptor space forms distinct and coherent functional clusters.

#### 2.7.2 Predictive modeling, validation suite architecture, and descriptor interpretability

To establish the practical utility of the refined descriptor set, a multiclass classification framework was implemented using multinomial Logistic Regression, configured with the default regularization strength (C = 1.0) and a maximum iteration threshold of max_iter = 1,000 to guarantee absolute numerical convergence. The optimization routine utilized the standard multi_class = “multinomial” solver parameterized within scikit-learn to directly compute cross-entropy minimization across all four categorical lineages simultaneously. This model was selected to maintain direct clinical interpretability, allowing quantitative mapping of descriptor contributions to subclass identity through model coefficients.

To evaluate both diagnostic sensitivity and baseline specificity under identical screening conditions, the baseline dataset, comprising a total of 548 structural variants (73 TEM, 70 SHV, 131 CTX-M targets, and 274 NON control entries), was partitioned into training and test segments using a sequence-aware strategy to minimize redundancy bias. This partition was executed at an approximate 70:30 ratio, allocating 384 variants for core model training (52 TEM, 48 SHV, 91 CTX-M, and 192 NON) and establishing a comprehensive independent test set (n = 164) held entirely separate from training.

To minimize homology-driven data leakage, partitioning was executed using a strict cluster-aware protocol at the subclass level. Because initial data curation isolated unique mutational singletons using a 100% sequence identity threshold, the maximum pairwise sequence identity across the train/test split boundary was strictly bound below 100%, ensuring no identical variants appear across the partitions. Furthermore, closely related nearest-neighbor variants and multi-sequence clusters were retained within a single data pool rather than being divided across splits. Further, a BLASTp analysis of all 164 validation structures against 384 training scaffolds revealed 88.50% average pairwise identity (96.11% coverage), indicating both sets comprise mutually exclusive sequence datasets. The independent test set was structured across two evaluation arms:

- Independent Positive Validation Arm (n = 82): Comprising active, experimentally characterized β-lactamases strictly withheld from model building, balanced across 21 TEM, 22 SHV, and 39 CTX-M variants to definitively evaluate classification sensitivity.
- Independent Negative Validation Arm (n = 82): Comprising a matched cohort of 82 non-target bacterial hydrolases (NON) entirely separate from the training matrix to baseline background false-positive rates.

Internal model robustness, parameter stability, and generalization across these primary data partitions were assessed using 10-fold repeated cross-validation executed exclusively within the training pool. The 164 sequences independent test set was completely segregated from all upstream data pipelines, serving as an untouched testing matrix that was not involved in descriptor extraction, feature selection, cross-validation tuning, or threshold optimization. In addition, standardized regression coefficients were extracted from the final trained architecture to quantify the relative contribution of each descriptor, thereby providing a transparent, linear representation of the biophysical determinants governing subclass classification.

To ensure absolute statistical integrity and eliminate any possibility of downstream data leakage, a strict computational firewall was enforced immediately following initial feature matrix compilation (Supplementary Figure S1). The sequence-aware data split was executed prior to any feature processing, standardization, or mathematical reduction, cleanly separating the training segment (n = 384) from the independent validation suite (n = 164). Feature scaling using StandardScaler was computed exclusively on the training matrix arrays via .fit_transform(), and the resulting sample mean and variance matrices were applied to the validation test arrays as static linear transformations via.transform(). Similarly, feature ranking operations (ANOVA, Random Forest importance, and SHAP feature attributions) and dimensionality compression via Principal Component Analysis (PCA) were strictly within the training pool boundaries. The independent validation profiles were completely insulated from these selection pipelines, undergoing simple spatial projection into the frozen training component coordinates without contributing to the underlying variance-covariance structures. This architecture ensures a zero-leakage pipeline configuration where validation metrics reflect true out-of-sample generalization power.

#### 2.7.3 Statistical validation of classification confidence

To ensure that subclass assignments reflect meaningful separation rather than marginal score differences, a *post hoc* statistical validation was performed on the test dataset. For each protein, subclass prediction scores were evaluated as a distribution, and the predicted class was required to demonstrate statistical significance relative to alternative classes using a Z-test (p < 0.05). Predictions were categorized into: (i) Significant classifications, where a clear dominant biophysical signature supports subclass assignment; and (ii) Ambiguous classifications, where competing subclass signatures indicate transitional or intermediate states. This framework provides an additional layer of confidence assessment, enabling identification of variants that occupy boundary regions within the functional landscape.

Collectively, this multi-tiered methodological framework integrates sequence curation, structural characterization, high-dimensional descriptor extraction, and interpretable modeling to establish a robust and biologically grounded representation of β-lactamase diversity. By systematically linking molecular signatures to structural descriptors, evolutionary context, and classification outcomes, the approach ensures that subsequent analyses are not only statistically rigorous but also mechanistically interpretable. This design facilitates direct translation of computational findings into pharmacologically relevant insights, particularly in understanding the structural determinants of substrate specificity and resistance evolution.

### 2.8 External validation dataset construction

To further examine the generalizability and specificity of the descriptor framework beyond the internally balanced training and control cohorts, an independent external validation dataset comprising 271 proteins was assembled from the UniProt database. Protein entries were selected after comprehensive manual cleaning and redundancy filtering. The final external dataset consisted of experimentally characterized and computationally annotated bacterial proteins spanning beta-lactamases, hydrolases, esterases, phosphatases, glycosylases, carboxypeptidases, lipases, peptidases, and related enzyme families.

In contrast to the structurally curated internal control set, this collection was intentionally designed as a high-stringency benchmark containing enzymes and proteins with potential sequence-, fold-, catalytic-, or annotation-level similarity to β-lactamases (AmpC family, OXA-type beta-lactamases, PER family enzymes, L1/L2 metallo-beta-lactamases, CphA metallo-beta-lactamases, Sme carbapenemases, PenA/PenP/PenPC family enzymes, and blaZ-associated beta-lactamases). Further a set of hard negative controls that are structurally or mechanistically related non-beta-lactamase enzymes belonging to hydrolase superfamilies, such as lipases and esterases, amidases, phosphatases, nucleotidases, glycosylases, peptidoglycan carboxypeptidases/PBPs, quorum-quenching lactonases, dehalogenases, ribonucleases, and DNA repair hydrolases. This composition enabled assessment of false-positive behavior against proteins sharing catalytic motifs or fold similarity with beta-lactamases. While the panel is highly challenging, it represents a selective rather than a fully exhaustive repository.

In terms of taxonomic diversity in the compiled external dataset, sequences originated from a broad range of bacterial taxa including: *Proteobacteria*, *Firmicutes*, *Actinobacteria*, *Bacteroidota*, *Cyanobacteria*, *Spirochaetota*, *Deinococcota*, *Thermotogota*, and *Mollicutes*. This taxonomic spread was included to reduce lineage bias and assess performance across phylogenetically distant organisms. The external dataset was used exclusively for independent validation. Performance on this dataset was used to estimate real-world robustness, especially the ability to detect diverse beta-lactamase subclasses, reject unrelated hydrolases, tolerate annotation heterogeneity, generalize across distant taxa.

### 2.9 Computational infrastructure, throughput, and pipeline scalability

To evaluate the operational feasibility of the 30-descriptor framework for high-throughput screening, a comprehensive computational profiling analysis was conducted. The pipeline architecture is structurally divided into two sequential phases: upstream structural coordinate generation and downstream feature extraction/inference. Downstream execution, comprising the computation of the 30 finalized multi-scale biophysical descriptors and model inference via the low-dimensional Logistic Regression classifier, requires less than 0.5 s per query on a standard desktop workstation equipped with a single-core Intel Core i7 CPU (2.4 GHz) and 16 GB RAM.

Because descriptor extraction and matrix operations scale linearly (O(N)), this phase is computationally lightweight, allowing high-throughput screening of pre-modeled sequences. The primary computational bottleneck resides in the upstream phase when processing completely uncatalogued variants lacking experimental structures in the Protein Data Bank (PDB). Generating accurate 3D atomic coordinates via AlphaFold3 (AF3) requires dedicated high-performance computing (HPC) infrastructure. Crucially, this structure-prediction step is an asynchronous and required exclusively for novel, non-redundant mutational variants to generate the baseline coordinate file. Once structural properties are locked, the downstream biophysical compression and subclass assignment scale efficiently, potentially offering application for integration into high-throughput genomic surveillance.

## 3 Results and discussion

### 3.1 Integrated dataset curation, taxonomic representation, and structural coverage

A multi-stage curation workflow was implemented to generate a high-confidence dataset of clinically relevant Class A β-lactamases (TEM, SHV, and CTX-M subclasses) suitable for downstream descriptor engineering and machine learning.

#### 3.1.1 Redundancy reduction preserves variant-level diversity

Cluster statistics demonstrated strong singleton enrichment across all subclasses, indicating genuine sequence diversity rather than repeated deposition artifacts. TEM contained 62 singleton clusters (84.9%), SHV contained 61 (87.1%), and CTX-M contained 107 (81.7%). Although some exact duplicate clusters were observed, most notably in TEM, most retained representatives were unique variants. Figure 1A depicts a violin plot of subclass-wise cluster-size distributions. The violin plots show the distribution of sequence cluster sizes for TEM, SHV, and CTX-M β-lactamase datasets following 100% identity dereplication. Width reflects density of clusters at a given size, while internal boxplots indicate median and interquartile range. CTX-M exhibited broader cluster-size dispersion, consistent with greater recent diversification and host dissemination, whereas SHV clusters were comparatively concentrated.

**FIGURE 1:**
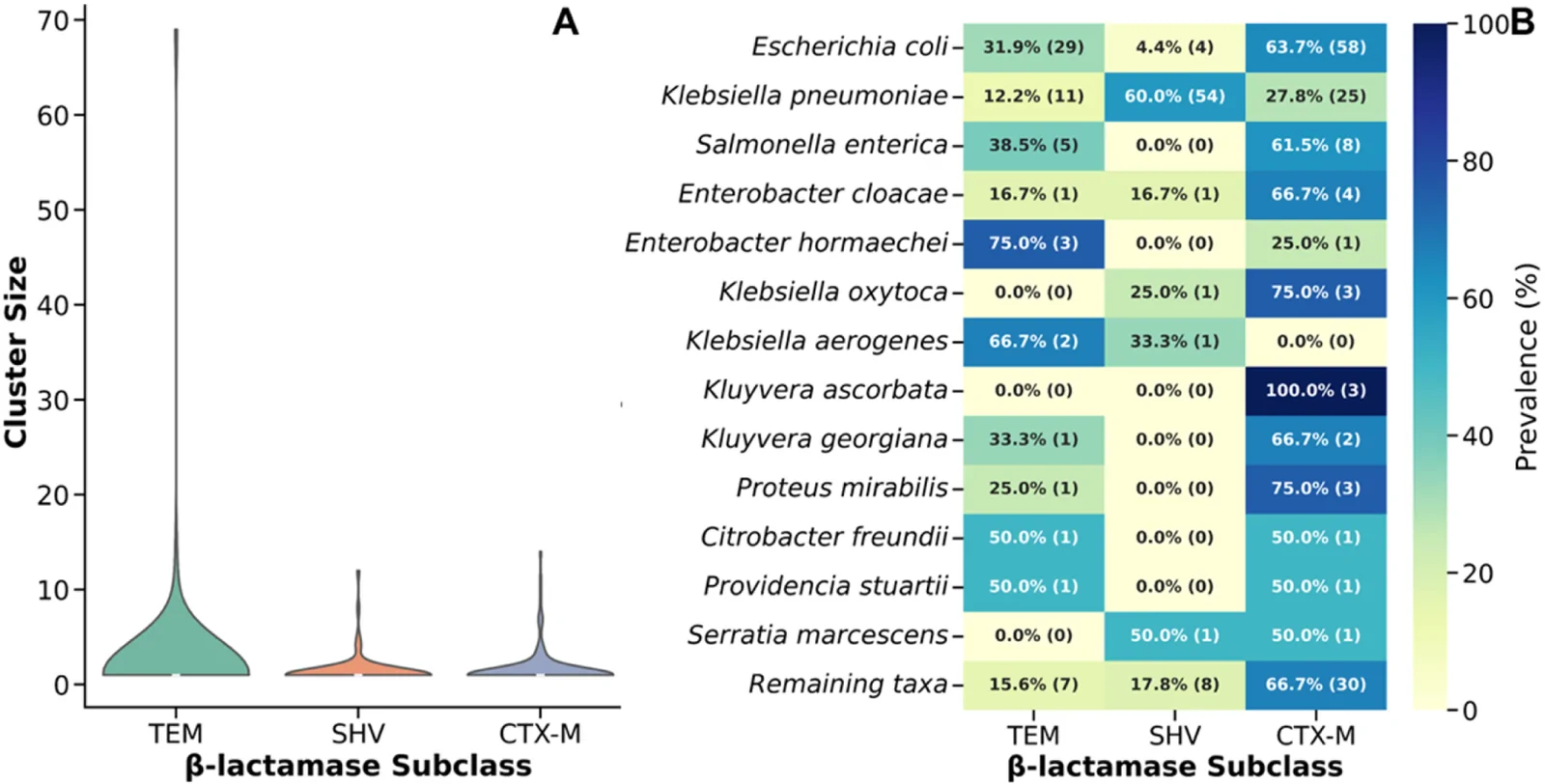
Sequence diversity and host-species structure of the curated class A β-lactamase dataset. **(A)** Violin plots show the distribution of sequence cluster sizes for TEM, SHV, and CTX-M β-lactamase datasets following 100% identity dereplication. **(B)** Heatmap showing the number of non-redundant sequences assigned to each host species across TEM, SHV, and CTX-M subclasses. Color intensity corresponds to entry count per species–subclass combination.

These observations are important in the context of resistance surveillance. Highly sampled variants often represent globally disseminated clinical lineages, whereas singleton variants may correspond to localized or emerging mutational events. Retaining both categories improves biological realism and strengthens classifier generalizability.

#### 3.1.2 Taxonomic structure reflects known epidemiology

Species-level composition of the non-redundant dataset closely recapitulates known host associations of major Class A β-lactamases. The TEM subset was dominated by *Escherichia coli* (n = 29), followed by *Klebsiella pneumoniae* (n = 11) and *Salmonella enterica* (n = 5), consistent with the long-standing association of TEM enzymes with plasmid-mediated dissemination in *Enterobacterales*. The SHV subset showed marked concentration within *K. pneumoniae* (n = 54 of 70), reinforcing the historical origin and ecological persistence of SHV scaffolds in *Klebsiella* species. In contrast, CTX-M displayed the broadest host range, led by *E. coli* (n = 58) and *K. pneumoniae* (n = 25), but also including *Salmonella*, *Enterobacter*, and multiple *Kluyvera* species. The presence of Kluyvera spp. is particularly informative, as chromosomal *Kluyvera* enzymes are widely recognized as ancestral progenitors of mobilized CTX-M families. Figure 1B represents a heatmap of species distribution across β-lactamase subclasses. The heatmap shows the number of non-redundant sequences assigned to each host species across TEM, SHV, and CTX-M subclasses. Color intensity corresponds to entry count per species–subclass combination. SHV sequences were strongly enriched in *K. pneumoniae*, TEM was dominated by *E. coli*, and CTX-M displayed the broadest host range, including multiple *Enterobacter*, *Salmonella*, and *Kluyvera* taxa.

Thus, the curated dataset not only preserves sequence diversity but also maintains authentic ecological and evolutionary patterns (Bush and Bradford, 2020; Doi et al., 2017). This is especially relevant for translational AI models intended for surveillance or One Health deployment, where taxonomic realism can materially affect external validity.

#### 3.1.3 External challenging validation dataset: structurally diverse mimics and functional decoys

The external dataset incorporated proteins from multiple mechanistic and structural classes. A substantial fraction consisted of non-target β-lactamases representing alternative Ambler classes, including AmpC-type cephalosporinases (Class C), OXA-type oxacillinases (Class D), and metallo-β-lactamases such as CphA, L1, L2, and DIM-like enzymes (Class B). Additional serine β-lactamases including PER-, SME-, PenA-, PenP-, and BlaZ-related proteins were also represented. Inclusion of these proteins created a stringent discrimination scenario in which the classifier was required to distinguish the focal TEM, SHV, and CTX-M subclasses from other *bona fide* β-lactamases with convergent resistance function but distinct structural and evolutionary origins (Bush and Bradford, 2020; Bush, 2018; Hall and Barlow, 2005).

A second major component of the dataset consisted of penicillin-binding proteins (PBPs), D,D-transpeptidases, and related peptidoglycan-modifying enzymes, including FtsI, PBP2, PbpA, AmpH, VanY, and multiple Dac-family carboxypeptidases. These proteins are particularly relevant to negative controls because they share ancestral relationships with serine β-lactamases and often conserve catalytic serine motifs associated with β-lactam interaction. Their inclusion therefore tested whether the descriptor space captured subclass-specific signatures rather than generic features of penicillin-recognizing enzymes. The dataset further included a broad range of hydrolytic enzymes from the α/β-hydrolase and metallohydrolase superfamilies, such as lipases, esterases, cutinases, haloalkane dehalogenases, phosphodiesterases, phosphatases, glyoxalase II proteins, undecaprenyl-diphosphatases, and acetylpolyamine amidohydrolases. These proteins were selected because they contain catalytic nucleophiles, metal-binding centers, or active-site cavities that may superficially resemble β-lactamase architectures. In addition, glycosidases and carbohydrate-active enzymes (e.g., β-hexosaminidases, xylanases, glucosaminidases, porphyranases), DNA repair enzymes (MutY, Fpg, Ung, Endonuclease IV/V), ribonucleases, and miscellaneous metabolic enzymes were incorporated to maximize biochemical and topological diversity (Bush, 2018; Ozhelvaci and Steczkiewicz, 2025; Lee et al., 2025). Table 3 outlines the functional composition of the external validation dataset.

**TABLE 3:** Functional composition of the external validation dataset (n = 271 proteins). The independent external validation dataset was assembled as a high-stringency benchmark comprising proteins with potential sequence-, structural-, catalytic-, or annotation-level similarity to β-lactamases. Entries were manually grouped into broad functional categories based on UniProt annotations and known biological roles.

Major functional category	Representative proteins/families	Biological relevance to benchmarking	Count*
Non-target β-lactamases (classes B, C, D and serine β-lactamases)	AmpC, OXA, CphA, L1, L2, DIM, PER-1, SME, PenA, PenP, PenPC BlaZ, BOR	Tests discrimination of target class A subclasses from other β-lactamases	85
Penicillin-binding proteins (PBPs), transpeptidases, carboxypeptidases	FtsI, PBP2, PbpA, PbpC, PbpG, PbpI, PbpX, AmpH, DacA/B/C/D/F, VanY	Evolutionarily related serine-active-site proteins interacting with β-lactams	38
Lipases, esterases, cutinases, depolymerases, dehalogenases	LipV, LipU, LipI, arylesterase, EstA, BioH, cutinase Est2, PhaZ, DCH, DhaA	α/β-hydrolase fold proteins with catalytic nucleophiles and ester-cleaving activity	27
Metallohydrolases/phosphatases/phosphodiesterases	GloB, SurE, SerB, SuhB, ApaH, AphA, CpdA, UPPP, PhnX	Tests confusion with metal-dependent catalytic pockets and active sites	31
Amidases/aminohydrolases/deaminases	AmiE, AnsA, AnsB, glutaminase-asparaginase	Catalytically diverse hydrolases with amide-bond processing functions	18
DNA repair/nuclease/RNA-processing enzymes	Ung, MutY, Fpg, RecU, Endonuclease IV/V, TatD, RNase III, RNase	Structurally unrelated but catalytic proteins expanding descriptor space	29
Glycosidases/carbohydrate-active enzymes	NagZ, xylanases, glucosaminidase LytG, β-galactosidase subunits	Diverse folds and substrate channels; tests structural generalization	16
Miscellaneous enzymes/other proteins	FolD, RsgA, AarA, YclE, YtnP, HdpA and other annotated proteins	Broadens biochemical and structural diversity	27
Total	​	​	271

* Counts are approximate category totals derived from UniProt annotation and may vary slightly depending on classification of multifunctional proteins.

While this validation panel is not fully exhaustive of all known non-target Ambler Class A variants, these specific enzyme families were intentionally prioritized to provide a balanced, highly challenging screening matrix (n = 271) that numerically scales with our target and baseline control datasets. By integrating diverse functional phenotypes, including carbapenemases (SME), extended-spectrum cephalosporinases (PER), and narrow-spectrum penicillinases (BlaZ), the external dataset establishes a challenging baseline to verify whether the 30-descriptor framework captures subclass-specific boundaries rather than generic Class A fold architectures. This selective yet rigorous arrangement evaluates model specificity against evolutionary neighbors that share overlapping core topologies but distinct resistance properties.

Taxonomically, the external benchmark spanned a wide phylogenetic range, including representatives from *Proteobacteria*, *Firmicutes*, *Actinobacteria*, *Bacteroidota*, *Cyanobacteria*, *Spirochaetota*, *Mollicutes*, thermophilic bacteria, and numerous environmental or marine lineages. The taxonomic diversity of the external validation dataset is summarized in Supplementary Table S3. The external validation dataset spans a broad phylogenetic range of bacterial taxa to minimize lineage-specific bias and ensure that predictive performance was not driven by narrow organismal composition. This broad organismal representation reduced lineage-specific composition bias and ensured that model performance was not driven by narrow taxonomic signals. For all proteins in the external dataset, experimentally determined structures were used whenever suitable high-quality entries were available. Sequences lacking reliable experimental structures were modeled using the standalone AlphaFold3 workflow under the same standardized conditions applied to the primary and internal control datasets (Abramson et al., 2024). This ensured methodological consistency during descriptor extraction and downstream comparative analyses.

Overall, the external dataset served as an adversarial validation layer positioned beyond conventional train-test benchmarking. By combining alternative β-lactamases, PBP-related proteins, diverse hydrolases, and phylogenetically heterogeneous enzymes, this dataset provided an assessment of whether the minimalist descriptor code captured the defining structural logic of Class A TEM, SHV, and CTX-M β-lactamases rather than broad enzymatic similarity alone.

#### 3.1.4 Pharmacological relevance of the unified structural framework

Across all datasets, the combined use of experimental structures and high-confidence AlphaFold3 models enabled extraction of a harmonized panel of structural and biochemical descriptors linked to catalytic pocket geometry, solvent accessibility, electrostatic organization, residue connectivity, and ligand-interaction potential. This complete structural framework was critical for subsequent identification of interpretable resistance signatures and for mapping molecular determinants relevant to inhibitor susceptibility. Collectively, these results establish a structurally robust foundation for mechanism-guided classification of β-lactamases and provide a scalable framework for computational pharmacology workflows targeting antibacterial resistance.

### 3.2 Evaluation of protein language model–derived latent representations

To determine whether modern protein language model (pLM) embeddings offer practical advantages for antimicrobial resistance profiling, latent representations generated from ProtT5 (sequence-derived) and ESM-3 (structure-aware multimodal) models were benchmarked against the curated biochemical and structural descriptor framework described in Section 2.5 (Elnaggar, 2021; Hayes et al., 2025). This analysis was undertaken to assess whether large-scale learned representations capture subclass-specific signatures relevant to differentiation of TEM, SHV, and CTX-M β-lactamases, which are among the most clinically significant Class A enzymes driving resistance to β-lactam antibiotics. From a translational perspective, improved subclass discrimination may support resistance surveillance, and prioritization of variants for experimental inhibitor characterization.

Overall, the pLM-derived descriptor spaces encoded biologically meaningful signals and accurately recapitulated known subclass relationships. However, they did not confer measurable predictive benefit over the reduced interpretable descriptor set. These findings indicate that, for this application, compact mechanistically relevant descriptors remain more suitable than high-dimensional latent embeddings for robust and explainable resistance modeling.

#### 3.2.1 Variance structure, compressibility, and internal redundancy

The evaluated latent spaces consisted of 1,024 dimensions for ProtT5, 1,536 dimensions for ESM-3, and 2,560 dimensions for the integrated representation formed by concatenation of both embeddings. Variance filtering detected no zero-variance descriptors in any dataset, indicating that all latent coordinates varied across proteins and therefore contained measurable sequence- or structure-associated signals (Figure 2A). Despite this apparent information richness, substantial redundancy was observed. Correlation analysis identified 514 highly correlated descriptor pairs (|r| > 0.90) in ProtT5, 558 in ESM-3, and 1,101 in the combined representation (Figure 2B), demonstrating that many embedding dimensions captured overlapping variation. Mean absolute pairwise correlations were moderate (0.437, 0.421, and 0.408 for ProtT5, ESM-3, and combined spaces, respectively), consistent with partially shared but non-identical encoded descriptors.

**FIGURE 2:**
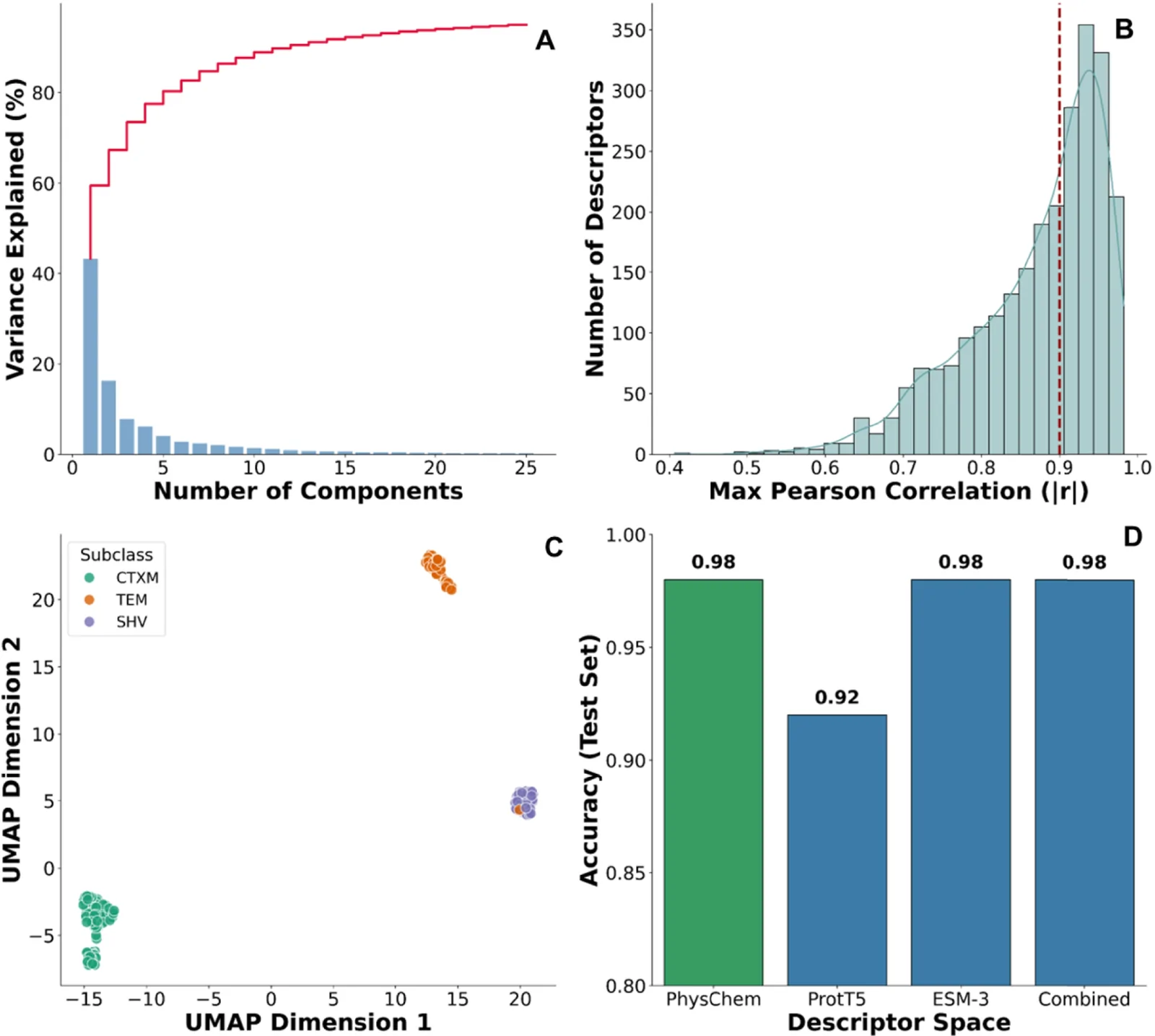
Dimensionality, descriptor, correlation, and performance metrics of integrated embeddings. **(A)** PCA scree plot illustrating the individual (bars) and cumulative (line) variance explained by the first 25 principal components; the gradual slope suggests high-dimensional complexity within the integrated descriptor space. **(B)** Distribution of maximum Pearson correlation coefficients (r) for each descriptor; the dashed line at 0.9 highlights the low proportion of highly redundant descriptors, confirming the relative orthogonality of the descriptor space. **(C)** UMAP projection of the latent space color-coded by β-lactamase subclass, demonstrating clear topological separation and clustering of functional groups. **(D)** Comparative model performance across different embedding strategies; while all methods achieve high accuracy, the combined model matches the peak performance of ESM-3 and physicochemical descriptors.

Principal component analysis further showed that all latent spaces were highly compressible (Figure 2A). In the integrated dataset, only 18 principal components were required to explain 90% of total variance, increasing to 36 and 101 components for 95% and 99% variance capture, respectively. Back-mapping of dominant components indicated that only 171, 279, and 464 descriptors materially contributed at these thresholds, representing a relatively small fraction of the original 2,560-dimensional input space. Similar trends were observed for ProtT5 and ESM-3 individually. These results indicate that although pLM embeddings contain informative biological patterns, their effective dimensionality is markedly lower than their nominal size. For β-lactamase subclass discrimination, much of the latent space therefore represents redundant or weakly informative variation rather than unique resistance-relevant signal. A quantitative summary is provided in Table 4.

**TABLE 4:** Quantitative evaluation of protein language model (pLM)-derived latent descriptor spaces generated from ProtT5, ESM-3, and their integrated representation. ProtT5 sequence embeddings (1,024 dimensions) and ESM-3 structure-aware embeddings (1,536 dimensions) were assessed individually and after concatenation into a unified 2,560-dimensional latent space. Reported metrics include total dimensionality, zero-variance descriptors, number of highly correlated descriptor pairs (|r| > 0.90), mean absolute pairwise correlation, and the number of principal components (PCs) required to explain 90%, 95%, and 99% of total variance. Values in parentheses denote the number of unique descriptors contributing substantially to the retained variance after back-mapping of dominant PCs.

Metric	ProtT5	ESM-3	Combined
Dimensions	1,024	1,536	2,560
Zero-variance descriptors	0	0	0
Highly correlated pairs (r > 0.9)	514	558	1,101
Mean abs cross-correlation	0.437	0.421	0.408
PCs (descriptors) at 90% variance	12 (111)	17 (157)	18 (171)
PCs (descriptors) at 95% variance	20 (163)	33 (239)	36 (279)
PCs (descriptors) at 99% variance	56 (252)	95 (387)	101 (464)
Accuracy on test dataset (after dropping correlated pairs + covering 90% variance)	0.915	0.988	0.988

^a^ Accuracy calculation: computed explicitly on the test dataset as the ratio of exact matching categorical assignments across all classes (TEM, SHV, CTX-M, and NON) to the total dataset (n = 164).

^b^ Prior to classification via multinomial logistic regression, feature vectors were standardized and compressed using Principal Component Analysis (PCA) to capture exactly 90% of the explained dataset variance. The resulting principal components (PCs) evaluated were 12 for ProtT5, 17 for ESM-3, and 18 for the Combined feature matrix.

#### 3.2.2 Complementarity between sequence- and structure-derived embeddings

To examine whether sequence-derived and structure-aware embeddings encode overlapping or complementary information, cross-modal correlation analysis was performed between ProtT5 and ESM-3 dimensions. The mean absolute correlation between modalities was low (0.28), and only 1.68% of descriptor pairs showed strong association (|r| > 0.70). This indicates that the two models capture largely distinct biological information.

This observation is consistent with model architecture. ProtT5 embeddings are primarily shaped by residue context, sequence grammar, and evolutionary constraints learned from large protein databases, whereas ESM-3 additionally incorporates structural context through coordinate-aware representations. Accordingly, one model emphasizes sequence conservation patterns, while the other captures aspects of three-dimensional organization that may influence catalytic behavior, substrate accommodation, and inhibitor interaction.

However, complementarity alone did not translate into superior predictive performance. In the present dataset, subclass divergence was already strongly encoded by lower-dimensional physicochemical and structural descriptors. Consequently, adding thousands of latent variables increased complexity without improving discrimination of clinically relevant β-lactamase groups.

#### 3.2.3 Intrinsic subclass separability and annotation resolution in latent space

Non-linear manifold learning using UMAP was applied to visualize the organization of TEM, SHV, and CTX-M variants within the integrated latent descriptor space (Figure 2C). Clear subclass-level partitioning was observed, with each lineage forming compact and largely non-overlapping clusters. This demonstrates that pLM embeddings preserve biologically meaningful sequence–structure relationships and recover the established architecture of major Class A β-lactamase families without explicit supervision.

Pairwise Euclidean distance analysis quantitatively supported these findings. Mean intra-subclass distances were 41.43 for TEM, 34.78 for SHV, and 46.30 for CTX-M, whereas mean inter-subclass distances were substantially larger: 71.78 for TEM versus SHV, 90.22 for TEM versus CTX-M, and 76.47 for SHV versus CTX-M. The separation between within-class compactness and between-class dispersion confirms that these enzymes occupy distinct regions of descriptor space. A limited number of proteins showed transitional clustering relative to their original annotations. Two notable examples were C5I571 (annotated as CTX-M but localized within the SHV cluster) and A0ABP7L288 (annotated as TEM but also positioned within the SHV region). Such cases may reflect annotation inconsistencies, borderline evolutionary placement, recombination events, or acquisition of mixed lineage-defining substitutions.

The case of C5I571 was especially informative. Its Euclidean distance to the SHV protein C5I552 was only 5.12, far below typical inter-subclass distances, indicating strong latent-space similarity. This proximity supports reassignment toward the SHV lineage and illustrates how descriptor-informed computational frameworks can help refine functional annotation of resistance enzymes. Such approaches may improve surveillance databases used for genomic resistance prediction and epidemiological tracking.

#### 3.2.4 Limited practical utility of pLM descriptors in the final predictive framework

Although ProtT5 and ESM-3 embeddings successfully captured subclass-defining biological signals, their inclusion did not improve downstream classification performance relative to the optimized classical descriptor framework. In practical terms, the high-dimensional latent spaces reproduced information already captured by interpretable variables linked to amino acid composition, physicochemical behavior, and structural organization. For a modest-sized dataset (n = 274), retention of 2,560 learned dimensions also increases the risk of overfitting, model instability, and reduced transparency. These considerations are especially relevant for translational antimicrobial resistance tools, where reproducibility, interpretability, and ease of deployment are critical for adoption in diagnostic or surveillance settings.

By contrast, the reduced descriptor framework achieved equivalent predictive performance using a compact descriptor set with direct mechanistic relevance to enzyme function and resistance phenotype. Such models are easier to validate biologically and more amenable to integration with inhibitor design workflows, resistance risk stratification, and molecular epidemiology pipelines. Despite informative latent organization (Figure 2C), inclusion of pLM-derived embeddings did not improve predictive performance over the optimized classical descriptor framework (Figure 2D).

To justify this architectural omission and map the precise predictive limits of these latent representations, an extensive multi-class ablation study was executed across the independent validation test dataset (n = 164), tracking macro- and weighted-average metrics across individual and concatenated configurations (Supplementary Table S4). When evaluated using their raw, full-dimensional feature matrices, the sequence-derived ProtT5 pipeline achieved a maximum weighted accuracy of 0.939 (MCC = 0.916), which shifted to 0.915 (MCC = 0.8716) upon compression to its 90% variance threshold. Conversely, the structure-aware multimodal ESM-3 platform exhibited a significantly higher predictive ceiling, reaching a global accuracy of 0.988 (Macro MCC = 0.981). Compressing the 978 ESM-3 dimensions down to its 12 principal components yielded an identical accuracy of 988 (Macro MCC = 0.981), confirming a high level of coordinate redundancy within the latent vector space.

Crucially, the concatenated integration of both models (Combined Original) offered no downstream performance benefits, yielding a lower macro accuracy of 0.976 (MCC = 0.962) before recovering to the 0.988 ceiling after PCA reduction. This mathematical plateau proves that combining sequence grammar and multimodal structure embeddings introduces extensive feature redundancy without extending subclass discrimination resolution.

Accordingly, consistent with the strategy outlined in Section 2.5, ProtT5 and ESM-3 embeddings were not incorporated into the final predictive framework. Subsequent analyses therefore focused on the refined interpretable descriptor space, which provided comparable accuracy while maximizing explainability, computational efficiency, and translational relevance for antimicrobial resistance research.

### 3.3 Comprehensive multi-dimensional biochemical and structural characterization

To establish an interpretable and mechanistically relevant framework for β-lactamase subclass discrimination, an integrated descriptor architecture was constructed by combining large-scale sequence-derived variables with structural, topological, and catalytic-site descriptors. The initial descriptor matrix comprised 9,314 variables spanning amino acid composition, dipeptide and tripeptide patterns, autocorrelation metrics, physicochemical distributions, residue network topology, solvent accessibility, and active-site microenvironment properties. This multi-scale representation was designed to capture both global determinants of protein stability and localized signatures governing β-lactam recognition, acylation efficiency, and resistance-associated catalytic behavior. Unlike high-dimensional latent embeddings (Section 3.4), these descriptors retain direct biochemical meaning, enabling resistance prediction models that are both accurate and mechanistically interpretable. A schematic overview of descriptor refinement and dimensionality reduction is shown in Figure 3A.

**FIGURE 3:**
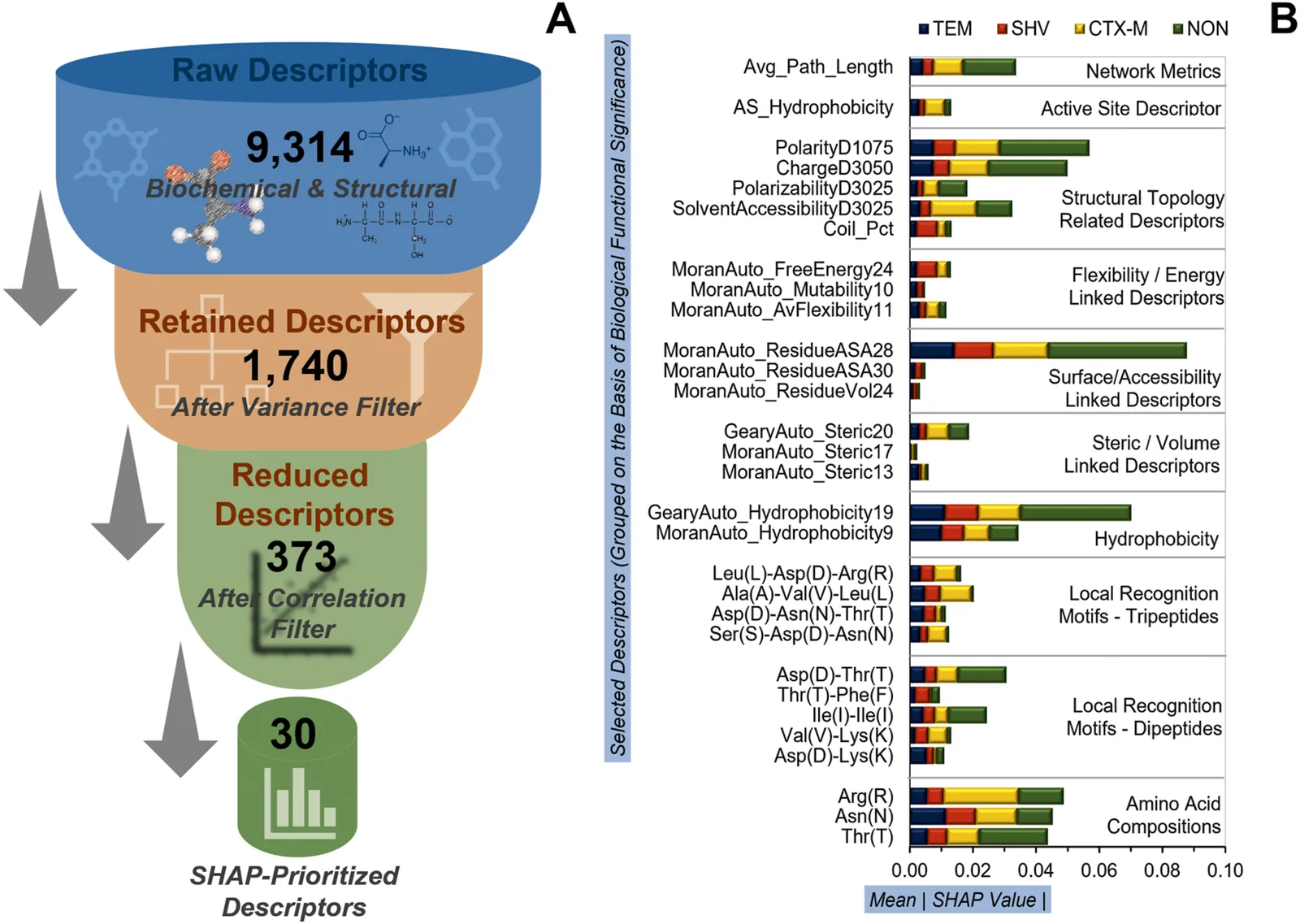
A depiction of multi-stage descriptor reduction workflow and composition of the final SHAP-selected descriptor space. **(A)** Funnel plot illustrating progressive dimensionality reduction of the initial biochemical and structural descriptor matrix used for β-lactamase subclass classification. **(B)** Bar chart showing the proportional distribution of descriptor classes represented within the final 30 SHAP-retained variables. The diverse composition indicates that β-lactamase subclass discrimination is governed by an integrated network of sequence, structural, and catalytic determinants rather than any single descriptor family.

#### 3.3.1 Descriptor variance analysis and dimensionality reduction

To optimize the descriptor space for downstream modeling, a variance-based filtering strategy was first applied to the 9,314 initial descriptors. A 25% occurrence threshold was used to retain only descriptors varying across at least 69 proteins, thereby excluding sparse or invariant variables with limited discriminatory utility. This step removed 7,574 descriptors (81.2%), most notably from higher-order compositional classes such as tripeptide composition, reflecting the strong sequence conservation that exists within major Class A β-lactamase lineages.

Subsequent correlation filtering (|r| > 0.90) was then applied to remove redundant variables and minimize multicollinearity. This process yielded a refined panel of 373 descriptors representing the non-redundant biochemical and structural signal space (Figure 3A). The retained variables encompassed residue accessibility, hydrophobicity distributions, flexibility indices, topological connectivity, active-site geometry, and long-range autocorrelation descriptors. These results indicate that only a relatively small subset of the original descriptor universe is required to capture subclass-specific resistance signatures. Importantly, the retained descriptors map directly to functional determinants of enzyme behavior, including substrate ingress, catalytic residue positioning, and stabilization of reaction intermediates.

#### 3.3.2 Principal component analysis and intrinsic dimensionality of the descriptor space

Principal component analysis demonstrated that the curated descriptor matrix was highly structured and compressible, indicating hierarchical organization of resistance-relevant biochemical information (Figure 3B). At lower dimensionality (∼50% cumulative variance), dominant contributors were long-range autocorrelation descriptors related to flexibility and free energy, suggesting that broad fold energetics account for a substantial fraction of observed variation.

At the 50% variance threshold, 157 descriptors distributed across 21 principal components were sufficient to represent the foundational signal space. These components were enriched for Geary, Moran, and Moreau-Broto autocorrelations, indicating that global spatial distribution of physicochemical properties forms the baseline architecture distinguishing β-lactamase subclasses. At intermediate resolution (∼75% variance), the model expanded to 285 descriptors across 63 principal components, incorporating localized sequence motifs and structural topology metrics. This transition suggests movement from generalized fold energetics toward lineage-specific determinants of substrate recognition and catalytic tuning. At higher resolution (90% variance), 336 descriptors across 119 principal components were required. These included active-site accessibility, steric distributions, and residue neighborhood metrics that likely govern fine-scale catalytic geometry and cephalosporin accommodation.

Collectively, this progression indicates that β-lactamase functional divergence is encoded through layered organization: global scaffold energetics first, followed by localized catalytic specialization. This architecture is consistent with the evolutionary adaptation of Class A enzymes toward altered substrate spectra and inhibitor susceptibility.

#### 3.3.3 Deconstructing descriptor contributions through SHAP analysis

To identify the most influential variables driving model decisions, SHAP (SHapley Additive exPlanations) analysis was performed on the optimized descriptor set. This yielded a minimal subset of 30 descriptors that collectively governed classification of TEM, SHV, CTX-M, and NON (Figure 3B). The top-ranking descriptors are summarized in Table 5. High-impact variables included MoranAuto_ResidueASA, polarity-associated descriptors, hydrophobicity autocorrelations, coil content, free-energy distributions, and residue volume metrics. These descriptors indicate that classification is determined by coordinated biophysical patterns rather than isolated sequence motifs. Mechanistically, the major descriptor groups correspond to three core functional axes:

- Substrate accessibility–Surface area and solvent exposure descriptors regulate entry and orientation of β-lactam substrates within the catalytic cleft.
- Electrostatic stabilization–Charge and polarity metrics influence stabilization of tetrahedral intermediates during acylation and deacylation.
- Structural communication–Network and autocorrelation descriptors reflect long-range residue coupling that preserves catalytic geometry during turnover.

**TABLE 5:** Summary of top-ranking descriptors identified through SHAP analysis that collectively define a minimal yet highly discriminative descriptor space for distinguishing TEM, SHV, and CTX-M β-lactamases from non-β-lactamase bacterial hydrolases (NON).

Subclass	Top descriptors	Biophysical significance
TEM	MoranAuto_Hydrophobicity9, Asn (N), DK, DNT, MoranAuto_AvFlexibility11, MoranAuto_Mutability10, MoranAuto_Steric13	Internal dynamics and packing Relies on specific hydrophobic lags and Asn-rich motifs to define the internal stability and packing of the TEM scaffold.
SHV	GearyAuto_Hydrophobicity19, MoranAuto_FreeEnergy24, Coil_Pct, TF, VK, MoranAuto_ResidueASA30, MoranAuto_ResidueVol24	Structural homology and flexibility Uses random coil content and long-range free energy correlations to resolve subtle structural dynamics distinct from TEM.
CTX-M	_SolventAccessibilityD3025, Arg (R), AVL, LDR, GearyAuto_Steric20, AS_Hydrophobicity, SDN, MoranAuto_Steric17	Energetic and hydrophobic profiling Driven by Arg-heavy motifs and high solvent accessibility to accommodate bulky expanded-spectrum substrates.
NON	_PolarityD1075, Thr (T), _ChargeD3050, Avg_Path_Length, MoranAuto_ResidueASA28, DT, II, _PolarizabilityD3025	Global scaffold constraints Differentiates general hydrolases via global polarity, charge distribution, and bulk surface area properties.

The SHAP summary profile (Figure 3B) demonstrates that resistance-associated subclass identity emerges from distributed interactions among sequence, structural, and microenvironmental descriptors rather than from any single dominant determinant.

#### 3.3.4 Minimal descriptor space and localized structural mapping of subclass differentiation

A refined set of only 30 SHAP-validated descriptors was sufficient to resolve subclass identity with high fidelity, demonstrating that β-lactamase divergence is tightly encoded within a compact, biologically meaningful descriptor space. To mathematically validate these descriptor profiles and eliminate mechanistic ambiguity, the highly ranked variables summarized in Table 5 were mapped directly onto the three-dimensional atomic coordinates of the prototype reference architectures: TEM-1 (PDB: 1ZG4), SHV-1 (PDB: 4JPM), and CTX-M-15 (PDB: 4HBT). Spatial auditing was concentrated across five important functional domains tracked using standard Ambler numbering: the primary active-site core (^70^STXK), the structural stabilization turn (^130^SDN), the terminal anchor strand (^234^KTG), the mobile catalytic Ω-loop (residues 163–178), and the hyper-variable R2 substrate-cleft flanking domain (residues 237–243). An all-against-all coordinate audit across these specific regions confirmed that each subclass exhibits a distinct descriptor signature that reflects real-world structural pharmacology boundaries rather than ungrounded mathematical correlations:

##### 3.3.4.1 TEM subclass validation (hydrophobic constraints and internal packing)

TEM enzymes were characterized by hydrophobic autocorrelations (MoranAuto_Hydrophobicity9) and Asn-centered motifs (Asn (N) enrichment). Coordinate back-mapping confirms that these descriptors stabilize the primary nucleophilic center (^70^STXK), which maps to the tightly packed Met68-Ser70-Thr71-Ser72-Lys73 sequence motif in TEM-1. These variables capture the highly conserved, rigid internal hydrophobic lags and specific asparagine networks that anchor the core α/β fold, safely locking the spatial positioning of Ser70 and neighboring catalytic residues to support stable acyl-enzyme formation and classical penicillinase activity.

##### 3.3.4.2 SHV subclass validation (loop content and conformational dispersion)

SHV enzymes showed strong enrichment for random coil content (Coil_Pct) and long-range free-energy variations (MoranAuto_FreeEnergy24). Localized DSSP back-mapping reveals that these feature variables track directly to the extended, non-helical loop networks flanking the catalytic center, specifically the high-mobility loop connecting the β3 and β4 structural strands. This signature accurately captures the subtle differences in loop flexibility and conformational dispersion that separate the SHV lineage from the more rigidly constrained TEM family, enabling adaptive substrate turnover and altered inhibitor responses through minor structural remodeling of the adjacent Ω-loop (Pro167-Asn170).

##### 3.3.4.3 CTX-M subclass validation (electrostatic tuning and solvent exposure)

CTX-M enzymes were explicitly defined by Arginine enrichment (Arg (R)), elevated solvent accessibility (_SolventAccessibilityD3025), and localized active-site hydrophobicity (AS_Hydrophobicity). Spatial auditing reveals that these variables physically cluster along the flexible entry hinges of the Ω-loop and the solvent-exposed margins of the R2 loop domain (Gly238-Arg244). Unlike the tightly packed, hydrophobic cleft boundaries seen in TEM-1 and SHV-1, the CTX-M-15 active cleft accommodates an accumulation of flexible, positively charged Arginine side-chains (such as Arg274 and Arg276) in close proximity to the ^234^KTG anchor motif. This unique architectural configuration generates a polar, highly solvent-accessible electrostatic funnel that lowers the steric and energetic barriers required to capture and hydrolyze bulky, expanded-spectrum oxyimino-cephalosporin substrates (e.g., cefotaxime).

##### 3.3.4.4 NON control subclass validation (global scaffold constraints)

The non-β-lactamase hydrolases (NON) were clearly distinguished by broader global polarity (_PolarityD1075), charge distributions (_ChargeD3050), and network topology signatures (Avg_Path_Length). These global variables reflect the absence of the localized, stereochemically precise active-site coordinates (such as the conserved ^130^SDN turn) required for active β-lactam ring coordination, separating broad background bacterial housekeeping enzymes from true resistance determinants.

Taken together, these findings demonstrate that subclass differentiation is driven not by major global fold rearrangements, but by a subtle, localized redistribution of electrostatic, steric, and solvent-accessibility descriptors within a conserved protein scaffold. This has direct translational relevance, as these descriptor-defined microenvironmental signatures bypass the limitations of sequence homology matching to provide an accurate, property-based foundation for next-generation inhibitor design, resistance surveillance, and functional annotation.

#### 3.3.5 Feature ablation optimization and comparative interpretability of the reduced framework

To mathematically validate the structural necessity of the finalized 30 descriptors panel and systematically map the performance-to-complexity trade-off curve, an ablation study was executed. The multinomial Logistic Regression classifier was evaluated iteratively across an incremental spectrum of descriptor counts, evaluating architectures constrained to the top 10, 15, 20, 25, 30, 35, 40, 45, and 50 highest-ranked biophysical and structural variables. Restricting the architecture to a minimal top 10 descriptor set severely degraded model resolution, generating 7 cross-class classification errors within the independent test dataset (comprising 3 CTX-M, 2 NON, 1 TEM, and 1 SHV mispredictions). This elevated error profile remained strictly unaltered when evaluating a 15 descriptors panel, before exhibiting incremental resolution gains at 20 descriptors (5 total misclassifications: 2 CTX-M, 1 NON, 1 TEM, and 1 SHV) and at 25 descriptors (3 total misclassifications: 2 CTX-M, 1 NON). A definitive optimization ceiling and performance plateau were successfully achieved at a threshold of exactly 30 descriptors. At this mathematical inflection point, prediction errors across the 164 sequence test dataset dropped to just 2 complex, low-margin transitional isolates (1 CTX-M to NON and 1 NON to CTX-M transition) while simultaneously achieving flawless 100% predictive fidelity for all canonical TEM and SHV lineages. Expanding the input feature space beyond this threshold to 35, 40, 45, or 50 descriptors yielded immediate diminishing returns, tracking an identical performance boundary of 2 misclassified edge cases. Consequently, the 30-descriptor panel was selected as the optimal mathematical elbow, maximizing subclass-specific classification accuracy while ensuring a minimized and parsimonious input space.

From an applied pharmacology perspective, the reduced descriptor space offers substantial advantages over black-box latent representations. The selected variables are computationally efficient, biologically interpretable, and directly linked to catalytic determinants of resistance. As a result, they provide a practical basis for predictive tools aimed at rapid annotation of newly identified β-lactamases, prioritization of inhibitor testing, and monitoring of resistance evolution. Accordingly, the final modeling framework was based on this optimized descriptor set rather than pLM embeddings, as it preserved predictive performance while maximizing explainability and translational utility.

#### 3.3.6 Topological validation of the minimal descriptor space by UMAP analysis

To determine whether the SHAP-prioritized minimal descriptor panel retained the subclass-discriminatory structure of the broader refined descriptor space, comparative UMAP analysis was performed using both the 373-descriptor dataset and the reduced 30-descriptor framework (Figures 4A–D). This analysis was designed to test whether substantial dimensionality reduction preserved the biochemical topology underlying TEM, SHV, CTX-M, and NON (non-β-lactamase hydrolase) classification.

**FIGURE 4:**
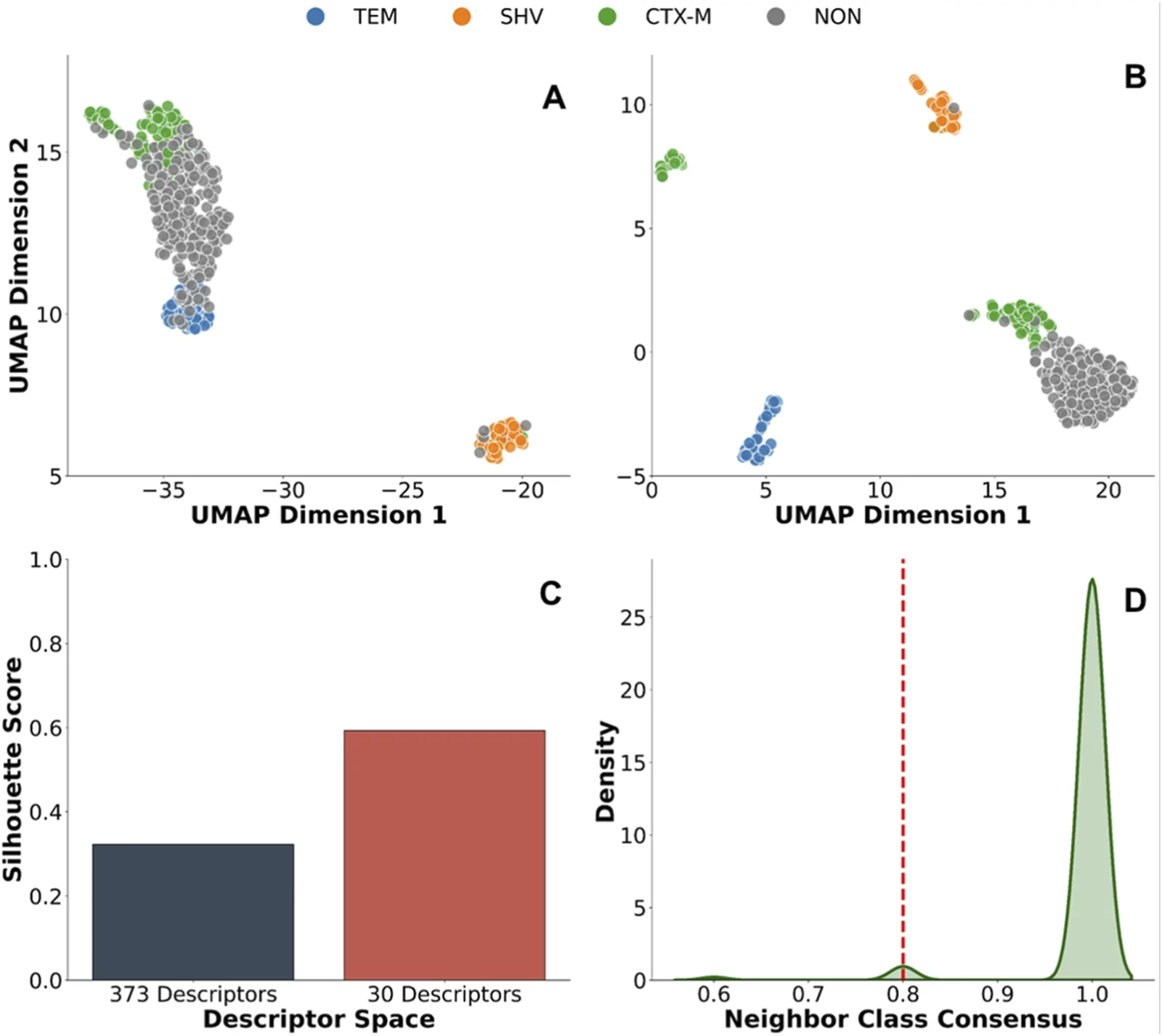
Topological preservation and clustering performance of the minimal descriptor framework following descriptor reduction. **(A)** UMAP projection of the refined 373-descriptor biochemical and structural descriptor space showing subclass-level organization of TEM, SHV, CTX-M, and non-β-lactamase hydrolases (NON). Distinct clustering indicates that the broader descriptor matrix captures lineage-specific resistance-associated molecular signatures. **(B)** UMAP projection of the SHAP-prioritized 30-descriptor minimal descriptor space. Clear retention of subclass segregation after dimensionality reduction demonstrates that a compact mechanistically interpretable descriptor panel preserves the essential discriminatory topology of the full refined dataset. CTX-M variants form two localized subclusters, suggesting internal subclass heterogeneity. **(C)** Silhouette score comparison between the 373-descriptor and 30-descriptor spaces. The increase in clustering coefficients from 0.32 to 0.59 after reduction indicates improved intra-class compactness and stronger inter-class separation in the minimal descriptor framework. **(D)** Kernel density distribution of five-nearest-neighbor subclass consensus within the 30-descriptor UMAP manifold. The dominant peak near 1.0 indicates that most proteins occupy highly homogeneous local subclass neighborhoods, whereas minor lower-consensus populations identify boundary or transitional variants with mixed descriptor characteristics.

Projection of the 373-descriptor descriptor space demonstrated clear organization of the four protein groups into largely discrete manifold regions (Figure 4A). SHV variants formed a compact and isolated cluster, whereas TEM proteins occupied a separate intermediate region. CTX-M variants are located to a distinct high-density zone positioned away from SHV and TEM. Non-β-lactamase hydrolases (NON) partially overlapped with the broader TEM/CTX-M region but remained topologically separable, indicating that although some global structural descriptors are shared across hydrolase scaffolds, subclass-specific catalytic signatures are retained within the descriptor space. Remarkably, the reduced 30-descriptor model preserved and further sharpened this class structure (Figure 4B). SHV and TEM variants remained strongly segregated, while NON formed a distinct peripheral cluster with minimal overlap. CTX-M proteins separated into two localized subclusters, suggesting the presence of internal heterogeneity within the expanded-spectrum lineage. This bifurcation may reflect known sequence diversification, differential substrate adaptation, or structurally distinct CTX-M subfamilies that remain unified at the subclass level but occupy related local manifolds.

Quantitative cluster analysis confirmed that dimensionality reduction improved descriptor-space resolution rather than degrading it. The silhouette coefficient increased from 0.32 in the 373-descriptor space to 0.59 in the 30-descriptor model (Figure 4C), representing a substantial gain in intra-class cohesion and inter-class separation. These results indicate that many variables retained in the larger descriptor matrix contributed noise or redundant variance, whereas the selected 30 descriptors concentrated the biologically relevant signals required for subclass discrimination. Local neighborhood analysis further supported the robustness of the reduced framework. Kernel density estimation of five-neighbor subclass consensus revealed a dominant peak at 1.0, indicating that most proteins were surrounded exclusively by members of the same subclass (Figure 4D). A secondary smaller peak near 0.8 suggested a limited subset of proteins positioned near cluster boundaries, while only a minor low-consensus population was observed near 0.6. These boundary cases likely represent transitional enzymes, annotation ambiguities, or proteins carrying mixed structural descriptors associated with broadened resistance phenotypes.

A more granular neighborhood-consensus analysis identified 23 proteins with mixed local subclass membership (consensus score < 1.0), representing candidates positioned near manifold boundaries or occupying intermediate descriptor states. A summary of these candidates is provided in Table 6. Most transitional cases were observed along the SHV–CTX-M and NON–SHV interfaces, suggesting that these groups share partially overlapping biochemical or structural characteristics within the reduced descriptor space. Several SHV variants (including A0A411G932, A0A386JTE5, A0A1Z1VQL0, Q79JA5, A0A5P8NAY5, and A0A286S471) showed directional proximity toward the CTX-M centroid, consistent with gradual divergence between narrow-spectrum and expanded-spectrum Class A β-lactamase phenotypes. Conversely, multiple non-β-lactamase localized toward the SHV region, indicating that certain non-β-lactamase scaffolds retain descriptor patterns resembling ancestral β-lactamase architectures. CTX-M variants A0ABN7C3G1, Q5MAF7, and A0AB35X3D9 displayed proximity toward the non-β-lactamases cluster, suggesting partial loss or redistribution of canonical subclass descriptors.

**TABLE 6:** Transitional variants identified from the 30-descriptor manifold using neighborhood-consensus and centroid proximity analysis. Variants with incomplete local class consensus (≤0.8) were considered boundary candidates. Lower distance-to-target values indicate stronger affinity toward the alternate class. Entries are arranged by biological relevance, prioritizing internal Class A β-lactamase shifts (TEM/SHV/CTX-M), followed by non-β-lactamase hydrolases (NON) exhibiting β-lactamase-like signatures, and CTX-M variants drifting toward NON space. These proteins represent candidates for annotation review, resistance surveillance, or functional validation.

Transition group	Variant	Distance to target	Group interpretation
SHV to CTX-M	A0A386JTE5	7.03	SHV variants exhibiting coordinated drift toward CTX-M descriptor space, consistent with acquisition of expanded-spectrum structural or catalytic signatures relevant to cephalosporin hydrolysis.
	A0A1Z1VQL0	7.08	
	A0A411G932	7.11	
	A0A286S471	7.80	
	C5I552	7.83	
	Q79JA5	7.85	
	A0A5P8NAY5	7.89	
CTX-M to SHV	C5I571	0.68	Strong internal reassignment toward SHV space indicates annotation inconsistency, lineage convergence, or shared scaffold signatures between CTX-M and SHV enzymes.
NON to SHV	I6WXK4	1.24	NON (non-β-lactamase hydrolases) showing SHV-like descriptor profiles, suggesting pre-adaptive scaffolds or latent resistance-compatible structural environments.
	Q09HD0	6.46	
	P94592	7.14	
	Q8ZRM2	7.77	
	Q840P9	7.90	
	Q9F663	7.97	
	P9WNH1	8.02	
	P52663	8.14	
	O34530	8.33	
	P52682	8.51	
NON to CTX-M	Q01166	5.28	Non-β-lactamase hydrolases approaching CTX-M space, potentially possessing pocket geometries or physicochemical traits permissive for extended-spectrum substrate recognition.
CTX-M to NON	Q5MAF7	2.43	Stronger ambiguity between CTX-M and NON space; may indicate borderline subclass assignment or functionally divergent CTX-M homologs.
	A0ABN7C3G1	2.72	
	A0A3Q8H7Q1	4.05	
	A0AB35X3D9	6.11	

The most notable boundary case was C5I571, annotated as CTX-M but positioned strongly toward the SHV cluster (distance = 0.684; consensus score = 0.8), closely paralleling the previously observed behavior of its near-identical counterpart C5I552. This result supports the possibility of annotation inconsistency or unresolved lineage assignment. Importantly, no substantial TEM-centered transitional population was observed, indicating that TEM variants occupy a comparatively stable and internally coherent region of descriptor space. These boundary proteins likely represent a mixture of mislabeled entries, evolutionary intermediates, or enzymes with hybrid catalytic microenvironments that blur conventional subclass boundaries. A focused evaluation of clinically relevant transitional candidates among TEM, SHV, and CTX-M variants is presented in Section 3.6.

Collectively, these findings demonstrate that the minimal 30-descriptor panel not only preserves the global subclass architecture present in the broader biochemical descriptor space, but also enhances separability, local classification confidence, and detection of borderline variants. From a translational perspective, this supports the use of compact, interpretable descriptor panels for rapid computational stratification of β-lactamases, surveillance of emerging resistance lineages, subclass reassignment of ambiguous annotations, and prioritization of variants for inhibitor susceptibility testing.

### 3.4 Transitional variants and boundary cases revealed by the minimal descriptor space

Although the 30-descriptor manifold produced strong global separation among TEM, SHV, CTX-M, and NON (non-β-lactamase) proteins (Section 3.3.6), local inspection of the UMAP topology revealed an additional layer of biological structure not evident from categorical labels alone. Specifically, a limited subset of proteins occupied positions near inter-cluster interfaces rather than within dense class cores. These proteins displayed partial descriptor compatibility with neighboring subclasses and therefore represent transitional variants or boundary cases within the reduced biophysical feature space. To systematically identify such proteins, two complementary criteria were applied: (i) local neighborhood consensus, measuring the proportion of nearest neighbors belonging to the annotated class, and (ii) centroid proximity analysis, quantifying relative distance to the centroid of alternate subclasses. Variants with incomplete neighborhood agreement (≤0.8) were designated boundary candidates, particularly when coupled with short distance-to-target values indicating attraction toward another class centroid. Using this framework, 23 proteins were identified as transitional cases (Table 6). These were distributed across four directional categories: CTX-M to SHV (n = 1), SHV to CTX-M (n = 7), NON to β-lactamase subclasses (n = 11), and CTX-M to NON (n = 4). This asymmetric pattern is biologically informative. It indicates that some subclass borders, especially between SHV and CTX-M, or between β-lactamases and broader hydrolase scaffolds, are relatively narrow in descriptor space, implying shared catalytic microenvironment properties despite divergent annotation histories. In contrast, TEM retained stronger internal cohesion with comparatively fewer boundary excursions.

Importantly, these proteins should not be interpreted merely as classification anomalies. Rather, they likely represent one or more of the following: (i) annotation inconsistencies in public databases, (ii) naturally occurring intermediates between recognized resistance lineages, (iii) convergent acquisition of similar catalytic physicochemistry, or (iv) enzymes with broadened or atypical substrate-recognition properties. Thus, the reduced descriptor model functions not only as a predictive framework, but also as a tool for biological discovery. The full set of candidates, including transition direction, annotation status, and proximity behavior, is summarized in Table 6.

#### 3.4.1 Intra-class A boundary shifts between SHV and CTX-M lineages

The most prominent internal Class A β-lactamase transitions involved directional movement between the SHV and CTX-M manifolds. These two clinically important ESBL-associated families are evolutionarily distinct, yet both hydrolyze expanded-spectrum cephalosporins and may therefore converge in descriptor space through shared active-site adaptations. The clearest example was C5I571, annotated as a CTX-M enzyme but positioned within the SHV-associated region of the manifold. This variant exhibited reduced local class consensus and strong proximity toward the SHV centroid, indicating that its integrated descriptor profile more closely resembles SHV enzymes than canonical CTX-M members. Such behavior may reflect historical recombination, lineage convergence, or incomplete annotation of an SHV-like scaffold labeled as CTX-M. Because SHV and CTX-M subclasses differ in inhibitor response patterns and substrate preference, these variants warrant direct biochemical reassessment.

Conversely, seven SHV-associated proteins shifted toward CTX-M space: A0A386JTE5, A0A1Z1VQL0, A0A411G932, A0A286S471, C5I552, Q79JA5, and A0A5P8NAY5. Notably, most derive from *Klebsiella pneumoniae* or related *Klebsiella*/*Raoultella* taxa, which are recognized reservoirs of SHV diversity. Their displacement toward CTX-M space suggests acquisition of physicochemical signatures commonly associated with CTX-M enzymes, potentially involving increased surface accessibility, altered polarity gradients, modified loop flexibility, or cavity remodeling favorable for oxyimino-cephalosporin accommodation. The presence of multiple SHV-to-CTX-M transitions, compared with only a single CTX-M-to-SHV case, suggests directional asymmetry in descriptor plasticity. This may indicate that subsets of SHV enzymes can more readily adopt CTX-M-like catalytic environments than the reverse. From a surveillance standpoint, such variants are especially relevant because they may represent precursor states from which broadened-spectrum phenotypes emerge under antibiotic selection.

#### 3.4.2 Non-target enzymes with β-lactamase-like biophysical signatures

A second major category involved proteins annotated outside the core target β-lactamase subclasses but positioned near resistance-enzyme clusters. These proteins belong to the NON reference group and include phosphatases, hydrolases, oxidoreductases, and distinct families of active β-lactamases. Their proximity to SHV or CTX-M space indicates significant overlap in the multi-dimensional descriptor combinations that define β-lactamase-like catalytic microenvironments.

Among these, ten proteins shifted toward the SHV cluster, a phenomenon driven by two distinct biochemical pathways: homologous structural conservation and convergent biophysical evolution. The first group comprises well-characterized Ambler Class A serine carbapenemases, including Q9F663 (KPC-2), Q09HD0 (GES-5), P52663 (NMC-A), and P52682 (SME-1). Because these enzymes share the identical primordial Class A core fold, active-site serine topologies, and structural loop configurations as the target SHV subclass, their close proximity within the descriptor manifold is highly expected and validates the framework’s baseline sensitivity to architectural homologs. Conversely, the second group captures a profound instance of structural convergence, exemplified by Q840P9 (VIM-7), a true Class B zinc-dependent metallo-β-lactamase (MBL). Despite lacking evolutionary lineage or sequence similarity to Class A serine enzymes, VIM-7 drifts toward the SHV boundary because the 30-descriptor pipeline detects convergent physical pocket traits that are required to coordinate and process the rigid β-lactam ring structure. The remaining non-β-lactamase proteins tracking near this zone, such as I6WXK4 (PtpB phosphatase), P94592 (YwpJ phosphatase), Q8ZRM2 (glyoxalase II), P9WNH1 (bromoperoxidase-like protein), and O34530 (RsgA GTPase), represent functional decoys whose electrostatic geometries or solvent-exposed clefts mimic these active resistance environments.

One additional protein, Q01166 from *Yersinia enterocolitica*, shifted toward the CTX-M cluster. Pairwise sequence analysis confirms that Q01166 shares a high sequence identity (92%) with the established Class A BlaA β-lactamase from the well-characterized y56 baseline strain. While *Kluyvera* species remain the primary confirmed evolutionary progenitors and mobile reservoirs for clinical *bla* CTX-M lineages, the role of *Yersinia* chromosomal lineages as direct origins of transferable resistance determinants remains unclear.

Collectively, these findings support the concept that broader hydrolase space contains latent structural backgrounds highly compatible with β-lactamase evolution. Under sufficient selective pressure, relatively few mutational steps may be required for these convergent scaffolds to acquire or broaden clinically relevant hydrolytic activity.

#### 3.4.3 CTX-M variants drifting toward non-β-lactamase space

Four CTX-M-annotated proteins localized toward the NON cluster: Q5MAF7, A0ABN7C3G1, A0A3Q8H7Q1, and A0AB35X3D9. These entries are largely unreviewed and originate from *K. pneumoniae*, *Klebsiella oxytoca*, *Kluyvera ascorbata*, and *Xenorhabdus taiwanensis*. Because *Kluyvera* species are recognized ancestral sources of CTX-M genes, displacement of some entries toward NON space may reflect ancestral scaffold states, incomplete specialization, truncated sequences, or overannotation of distant homologs as functional CTX-M enzymes. Alternatively, these proteins may encode weak or atypical β-lactamases with reduced catalytic efficiency relative to clinically optimized CTX-M variants.

This observation emphasizes that subclass labels in sequence databases do not guarantee equivalent biochemical behavior. Descriptor-space placement therefore provides a useful orthogonal filter for prioritizing questionable or atypical annotations.

#### 3.4.4 Biological and translational significance of transitional variants

The transitional proteins identified here demonstrate that β-lactamase evolution is better represented as a continuous manifold than as rigid subclass compartments. TEM, SHV, and CTX-M remain strongly separable at the population level, yet local border regions contain proteins occupying intermediate physicochemical states. Such proteins are of high translational interest because they may represent: (i) Emerging resistance precursors capable of acquiring expanded-spectrum activity, (ii) Misannotated database entries requiring curation review, (iii) Convergent scaffolds independently evolving β-lactamase-like catalytic environments, and (iv) Atypical enzymes with altered inhibitor susceptibility or substrate range.

Accordingly, candidates listed in Table 6 should be prioritized for downstream validation, including substrate hydrolysis assays, kinetic profiling, inhibitor susceptibility testing, and genomic surveillance in clinical isolates. Overall, this analysis demonstrates that the 30-descriptor model is not merely a classifier. It is an interpretable landscape-mapping framework capable of detecting evolutionary trajectories, hidden functional proximity, and early warning signals of future resistance diversification.

### 3.5 Predictive performance, statistical confidence, and model interpretability of the minimal descriptor framework

To assess the translational utility of the SHAP-prioritized 30-descriptor panel, a multiclass Logistic Regression (LR) classifier was developed to assign proteins into four clinically relevant categories: TEM, SHV, CTX-M, and NON (non-β-lactamase hydrolases). These subclasses were selected because accurate discrimination among major Class A β-lactamase families is essential for antimicrobial resistance surveillance, lineage tracking, resistance annotation, and prioritization of enzymes for inhibitor susceptibility studies. In addition to conventional predictive metrics, classifier outputs were statistically calibrated using score-derived Z-scores and p-values, enabling quantitative assessment of prediction confidence. Figure 5 summarizes the relationship between model scores, subclass assignments, and statistical significance.

**FIGURE 5:**
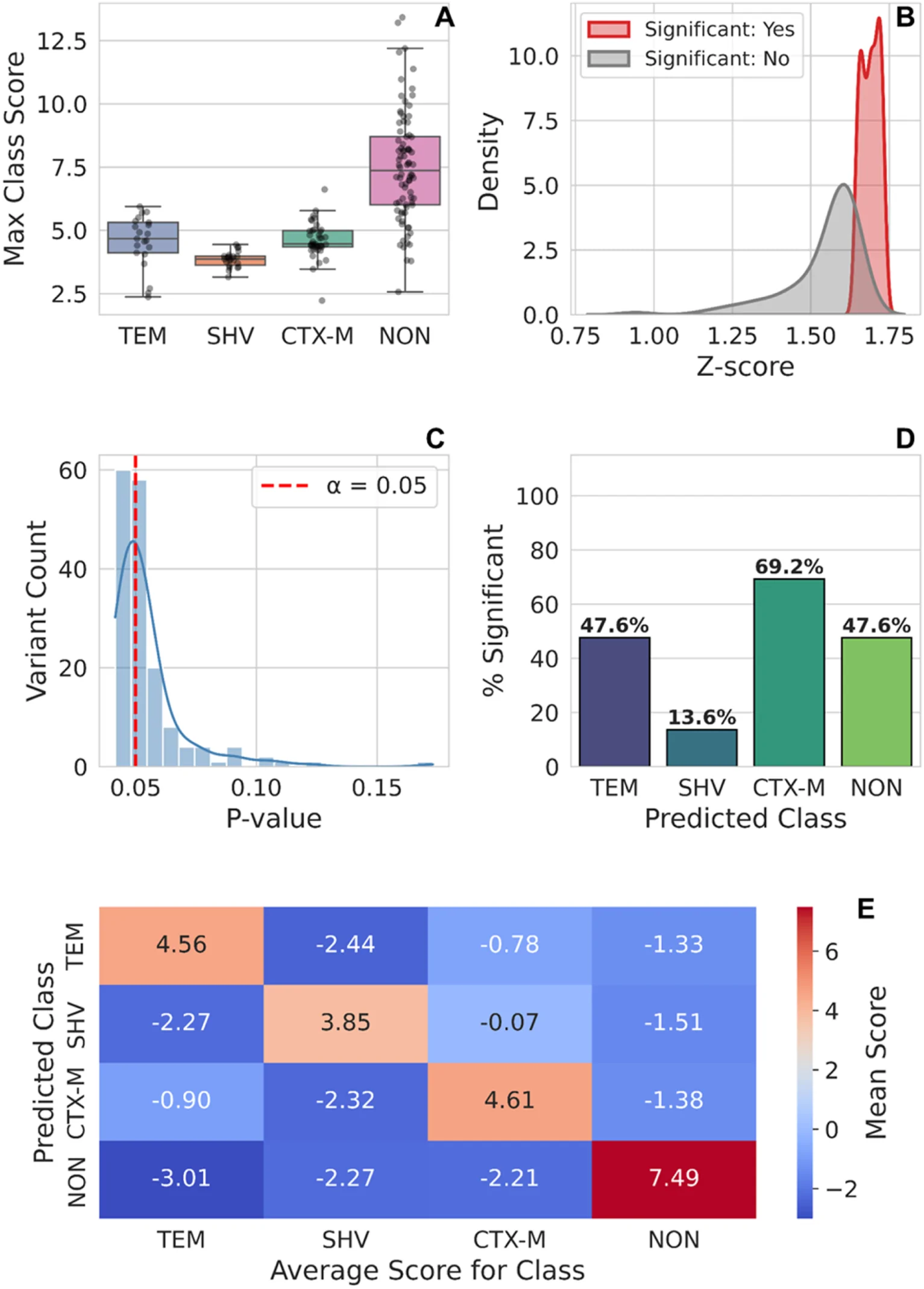
Statistical confidence and score-distribution analysis of subclass predictions generated from the test dataset. Five complementary panels summarize the relationship between classifier output scores, statistical significance, and predicted β-lactamase subclass assignments (TEM, SHV, CTX-M, and NON). **(A)** Distribution of winning prediction scores across predicted subclasses. Boxplots show the maximum subclass score assigned to each protein sequence, grouped by predicted subclass. Each point represents an individual sequence overlaid as a jittered dot. Boxes indicate the interquartile range (IQR), center lines indicate the median, and whiskers represent the spread of the non-outlier data. Higher winning scores indicate stronger classifier preference for the assigned subclass. This panel compares confidence magnitude among the four predicted categories. **(B)** Kernel density distribution of Z-scores stratified by statistical significance. Smoothed density curves depict the distribution of Z-scores for predictions labeled statistically significant versus non-significant. Significant predictions are expected to cluster at higher positive Z-scores, whereas non-significant predictions are concentrated at lower values. Separation between the two curves illustrates the discriminative capacity of the statistical confidence framework. **(C)** Histogram and kernel density estimate of P-values calculated for all predictions. The red dashed vertical line marks the conventional significance threshold (α = 0.05). Predictions left of this threshold are considered statistically significant. Enrichment of values below 0.05 indicates a substantial proportion of confident subclass assignments. **(D)** Percentage of statistically significant predictions by predicted subclass. Bar plot showing the proportion of predictions within each subclass category that achieved statistical significance (P < 0.05). Values above bars indicate exact percentages. This panel highlights differences in confidence calibration across predicted classes and reveals whether certain subclasses are classified with greater statistical certainty than others. **(E)** Mean subclass score matrix by predicted subclass. Heatmap displaying the average raw classifier scores for each predicted subclass (rows) against all four subclass scoring models (columns: TEM, SHV, CTX-M, NON). Warmer colors indicate higher mean scores, and cooler colors indicate lower mean scores. Correctly classified groups are expected to show elevated diagonal values, where the predicted subclass corresponds to its own scoring column. Off-diagonal signals indicate score overlaps or residual similarity between subclasses.

#### 3.5.1 Robust predictive confidence and comparative benchmarking across β-lactamase subclasses

The maximal subclass score assigned to each sequence was consistently elevated across correctly predicted subclasses, indicating strong separation between the assigned label and competing class scores. As shown in Figure 5A, the distributions of winning scores were tightly clustered within each predicted class, with relatively high medians and limited dispersion. This robust class preference was systematically quantified using both internal cross-validation and out-of-sample testing parameters (Table 7).

**TABLE 7:** Detailed lineage performance metrics across internal 10-fold cross-validation and the independent test dataset.

Lineage	Sensitivity	Specificity	Precision	F1-score	MCC
Part A: 10-fold cross-validation (mean ± SD) (global accuracy: 0.994 ± 0.011)					
CTX-M	0.990 ± 0.031	0.996 ± 0.010	0.989 ± 0.031	0.989 ± 0.023	0.986 ± 0.029
SHV	0.990 ± 0.100	0.997 ± 0.009	0.972 ± 0.112	0.980 ± 0.103	0.979 ± 0.104
TEM	1.000 ± 0.000	0.999 ± 0.003	0.998 ± 0.014	0.999 ± 0.007	0.999 ± 0.008
NON	0.994 ± 0.016	1.000 ± 0.000	1.000 ± 0.000	0.997 ± 0.008	0.994 ± 0.016
Part B: independent test dataset (global accuracy: 0.988)					
CTX-M	0.974	0.992	0.974	0.974	0.966
SHV	1.000	1.000	1.000	1.000	1.000
TEM	1.000	1.000	1.000	1.000	1.000
NON	0.987	0.987	0.987	0.987	0.975

Internal 10-fold cross-validation demonstrated architectural stability across the distinct data training folds, maintaining an overall global accuracy of 0.994 ± 0.011 and a macro-averaged Matthews Correlation Coefficient (MCC) of 0.992 ± 0.017. This high internal precision successfully generalized to the completely withheld independent validation test set (n = 164), tracking an overall global test accuracy of 0.988 and a macro specificity ceiling of 0.995. Lineage-specific evaluation showed perfect sensitivity and precision (1.0000) for all canonical TEM and SHV test variants, with zero errors crossing their split boundaries. Minor classification drift was restricted to just two complex edge cases across the entire independent validation suite: one characterized by a latent CTX-M to NON transition (A0ABN7C3G1) and another exhibiting a NON to CTX-M transition (Q09HD0). This pattern demonstrates that the reduced descriptor framework yields exceptionally stable class boundaries rather than marginal or ambiguous assignments. Among predicted subclasses, TEM, SHV, and CTX-M all exhibited strong score distributions, while the non-β-Lactamase group also maintained high confidence despite representing a broader non-resistance hydrolase background.

To contextualize these findings within existing diagnostic workflows, we conducted an independent benchmarking study against four industry-standard annotation platforms: AMRFinder+ (Feldgarden et al., 2022), CARD (RGI) (Alcock et al., 2023), DeepARG (Arango-Argoty et al., 2018), and PLM-ARG (Wu et al., 2023) (Supplementary Table S5). While these established platforms perform exceptionally well on traditional reference sequences, they remain intrinsically dependent on literal database string matches or consensus alignment footprints. When challenged with localized point mutations, evolutionary boundary spaces, or divergent environmental hydrolases, these sequence-dependent architectures create an analytical blind spot. To preserve precision, traditional platforms frequently default to broad, non-specific annotations (such as generic BETA designations), which limits their subclass sensitivity (90.24%–92.68%) and compromises family screening specificity (93.90%–97.56%). In contrast, our approach functions as an entirely alignment-free property classifier. By operating directly on a minimized, 30-dimensional landscape of multi-scale biophysical and geometric pocket traits, the model is decoupled from the rigid requirement of global sequence homology thresholds. This allows it to learn the actual physical rules governing the active site microenvironment, achieving superior classification fidelity (98.78% accuracy, 99.02% sensitivity, and 98.39% specificity). Consequently, when an emerging variant or a highly divergent environmental ortholog accumulates significant mutations that disrupt literal sequence alignment, our model bypasses the text-level changes to successfully recognize the underlying physical traits required for β-lactam coordination and hydrolysis. The ability to generate high-confidence, precise assignments across all four categories—without a dependency on global identity thresholds—shifts the surveillance paradigm from passive database matching to proactive resistance profiling. This provides a scalable, biochemically interpretable mechanism capable of monitoring and flagging novel, uncatalogued β-lactamases hidden within vast genomic and metagenomic surveillance datasets.

#### 3.5.2 Statistical validation of prediction confidence

To determine whether model assignments were statistically meaningful rather than numerically maximal by chance alone, Z-score normalization and associated empirical P-values were calculated for each prediction. The resulting distributions showed clear stratification between statistically significant and non-significant calls. As shown in Figure 5B, predictions classified as significant were concentrated at substantially higher Z-scores than non-significant predictions, indicating that standardized score separation captures prediction certainty. Complementing this result, the global P-value distribution (Figure 5C) showed marked enrichment below the conventional significance threshold of α = 0.05, demonstrating that most predictions were supported by statistically non-random score dominance. Notably, the two transitional variants, A0ABN7C3G1 (CTX-M to NON at p = 0.123) and Q09HD0 (NON to CTX-M at 0.051), were found to be statistically non-significant with p > 0.05. Together, these findings indicate that the LR classifier not only produces accurate subclass labels but also provides a quantitative confidence framework suitable for downstream decision-making. This is especially relevant for resistance surveillance pipelines, where uncertain calls may require secondary validation.

#### 3.5.3 Subclass-specific reliability of predictions

The proportion of statistically significant predictions varied across subclasses, indicating differences in descriptor cohesion, within-class heterogeneity, and the degree of score separation achieved by the classifier. As shown in Figure 5D, the highest rate of significant predictions was observed for CTX-M, where 27 of 39 predictions (69.2%) met the statistical significance threshold. This strong performance suggests that CTX-M enzymes retain a sufficiently coherent descriptor signature despite their recognized evolutionary diversity.

The TEM subclass showed an intermediate level of confidence, with 10 of 21 predictions (47.6%) classified as statistically significant. This result indicates moderate but reliable subclass separability, consistent with the conserved biochemical framework of TEM enzymes while allowing some overlap with related Class A β-lactamases. Predictions assigned to the NON class also demonstrated substantial reliability, with 39 of 82 predictions (47.6%) achieving significance. Given that this category comprises diverse non-β-lactamase hydrolases rather than a single evolutionary lineage, this level of significance is notable and indicates that the model effectively distinguishes general hydrolases from resistance-associated enzymes. This is operationally important because false-positive annotation of environmental or housekeeping enzymes as β-lactamases can distort resistance surveillance datasets.

Crucially, this 47.6% high-confidence significance rate for the NON cohort represents a deliberate and protective statistical asset rather than a functional limitation of the triage pipeline. In large-scale environmental genomic surveillance, background noise from uncharacterized bacterial hydrolases frequently mimics baseline biophysical traits of active resistance scaffolds. If a computational model forces a high-confidence categorical prediction on these ambiguous, low-margin sequence inputs, it inevitably introduces false-positive artifacts that artificially inflate regional resistome profiles. To circumvent this, our framework implements a highly conservative Z-score significance calibration that acts as a robust statistical safety net. While the underlying logistic model successfully rejects 81 NON sequences at the categorical level, the Z-score significance filter intentionally designates the remaining 52.4% of lower-margin background proteins as unresolved boundary states. By channeling ambiguous structural profiles into an intermediate, low-confidence review category instead of forcing a specific resistance assignment, the pipeline shields surveillance data from false-positive accumulation. Furthermore, this operational threshold can be dynamically customized based on user constraints: it can be tuned to maximum specificity (high Z-score stringency) to isolate pristine reference datasets or shifted toward maximum sensitivity (lower Z-score thresholds) for open-ended discovery campaigns aimed at capturing highly divergent, emerging target variants. The lowest significance rate was observed for SHV, where only 3 of 22 predictions (13.6%) were statistically significant. This reduced confidence may reflect close biochemical similarity between SHV and other Class A β-lactamase families, particularly TEM, leading to narrower score margins despite correct categorical assignment. Such behavior suggests that SHV proteins occupy a more overlapping region of descriptor space rather than representing model failure.

This highly variable subclass significance distribution represents a direct mathematical consequence of row-wise Z-score confidence modeling constrained within a closed four-class probability array. Because model-derived probabilities are normalized to sum to unity, a completely unambiguous assignment vector (i.e., [1.0, 0.0, 0.0, 0.0]) dictates a fixed mathematical Z-score ceiling of exactly 1.73205. Passed through a right-tailed normal cumulative distribution function (CDF), this ultimate statistical boundary yields an empirical floor p-value of 0.0416. Because this optimal cutoff sits immediately adjacent to the standard α = 0.05 significance threshold, any minor cross-class probability division will compress the Z-score below 1.645 and automatically relegate the sequence to a statistically non-significant designation. This mathematical ceiling explains why the distinct biophysical features of the CTX-M subclass map to a 69.2% significance rate, while the high sequence identity and core fold homology shared between TEM and SHV lineages suppress the latter. When processing an SHV variant, the classifier correctly assigns the lineage but routinely allocates a minor fractional probability to the neighboring TEM class (e.g., scoring an array like [0.85 SHV, 0.15 TEM]). This minor divergence pulls the row-wise Z-scores into a constrained window between 1.55 and 1.63 (as seen across the raw validation arrays), shifting their resulting p-values slightly above the alpha threshold (ranging from 0.051 to 0.059). Rather than indicating poor calibration or inappropriate testing, this conservative behavior highlights the biological reality of intra-family structural convergence and functions as a sensitive screening filter for highlighting evolutionary transition zones.

A key source of epistemic uncertainty in our framework stems from the intrinsic dynamic flexibility of the β-lactamase scaffold, which cannot be completely captured by static structural coordinates (Kaushik and Re, 2026; Gniadkowski, 2008; Gobeil et al., 2019). In Ambler Class A β-lactamases, the active-site pocket is flanked by highly mobile loops, such as the Ω-loop (Ambler residues 163–178) and the flexible loop connecting the β3 and β4 strands, which undergo structural transitions to accommodate and hydrolyze diverse substrates. Whether extracting coordinates from static X-ray crystal structures in the PDB or utilizing predicted conformational snapshots from AlphaFold3, minor structural variations in these loops alter local geometric metrics. These include solvent-accessible surface area (SASA), pocket volume, and electrostatic network configurations. Because our framework relies on these 3D properties, localized structural flexibility introduces variance into the feature extraction process. This variance shifts complex transitional variants or highly mutated environmental orthologs into close boundary zones along the UMAP manifold. This fluid biophysical landscape directly accounts for the two misclassifications observed in our validation dataset (A0ABN7C3G1 and Q09HD0). In these cases, specific mutations in dynamic regions mimic the physical profile of neighboring subclasses. Acknowledging this loop flexibility highlights that borderline model predictions are often not random computational errors, but rather reflections of true structural and evolutionary convergence across the TEM, SHV, and CTX-M landscape.

Overall, subclass-specific significance rates reveal that statistical confidence is not uniform across categories but instead reflects the intrinsic separability of each lineage within the reduced biochemical descriptor space as depicted in Figure 5D. High-confidence CTX-M predictions, moderate TEM and non-β-lactamase performance, and lower SHV significance collectively illustrate the biological complexity captured by the model.

#### 3.5.4 Descriptor-level logic and linear interpretability

Because the classifier is linear, subclass discrimination arises from additive combinations of interpretable biochemical descriptors rather than opaque nonlinear interactions. This is advantageous for translational deployment because model behavior can be directly related to underlying physicochemical properties.

The mean subclass score matrix shown in Figure 5E further supports this interpretation. Strong diagonal dominance was observed, whereby proteins predicted as TEM, SHV, CTX-M, or NON received their highest average scores from the corresponding subclass model. Off-diagonal values were comparatively lower, indicating limited cross-class confusion and biologically coherent score partitioning.

This structure implies that the major β-lactamase lineages occupy distinct yet interpretable regions of descriptor space. Subclass identity therefore appears encoded through combinations of polarity, residue composition, motif frequency, flexibility, solvent accessibility, and energetic autocorrelation descriptors rather than requiring complex hidden representations.

#### 3.5.5 Translational implications for resistance surveillance and therapeutic development

Collectively, Figure 5 demonstrates that the minimal descriptor LR framework provides three clinically valuable outputs simultaneously: accurate subclass assignment, quantified statistical confidence, and mechanistically interpretable score behavior. This has several practical implications:

- Rapid surveillance: newly sequenced enzymes can be assigned to major resistance families with associated confidence estimates.
- Improved annotation: uncertain or low-significance predictions can be flagged for manual review or secondary structural analysis.
- Therapeutic prioritization: reliable subclass calls can help in prioritization of variants for experimental inhibitor characterization.
- Explainable drug discovery support: descriptor-level separation reveals physicochemical traits relevant to future subclass-aware inhibitor design.

Accordingly, the SHAP-prioritized 30-descriptor framework functions not merely as a predictive model, but as a scalable and explainable platform for computational support of antimicrobial resistance monitoring, and precision therapeutic workflows.

### 3.6 External validation of the 30-descriptor model on a diverse independent dataset

To determine whether the SHAP-prioritized 30-descriptor model retained predictive utility beyond the internal benchmark set, an external validation analysis was conducted using 271 independent protein sequences that were not used during feature selection, model training, hyperparameter tuning, or internal performance assessment. All proteins in this challenge dataset were labeled as NON, representing enzymes outside the canonical TEM, SHV, and CTX-M β-lactamase subclasses. The external panel was intentionally assembled to provide a stringent specificity test, because many environmental hydrolases, cell-wall remodeling enzymes, and broad serine-active proteins can share partial structural or physicochemical similarities with resistance-associated Class A β-lactamases (Tehrani et al., 2018; Elnaggar, 2021; Hayes et al., 2025; Healy and McInnes, 2024). Under real surveillance conditions, such proteins are common sources of false-positive annotation. Accordingly, successful performance on this dataset provides a practical measure of whether the reduced descriptor panel can discriminate clinically relevant β-lactamase signatures from broader background enzyme diversity.

Despite the deliberate difficulty of the benchmark, the classifier preserved strong discrimination on previously unseen proteins and correctly recognized most sequences as NON. Misclassifications were infrequent and biologically structured rather than random, indicating that the descriptor manifold generalized outside the discovery dataset. Overall performance metrics, subclass-specific false-positive distributions, and prediction confidence trends are summarized in Figure 6. Further, Supplementary Table S6 summarizes the variants in the external dataset that reflected the descriptors tendencies towards Class A β-lactamases of TEM, SHV, or CTX-M subclasses.

**FIGURE 6:**
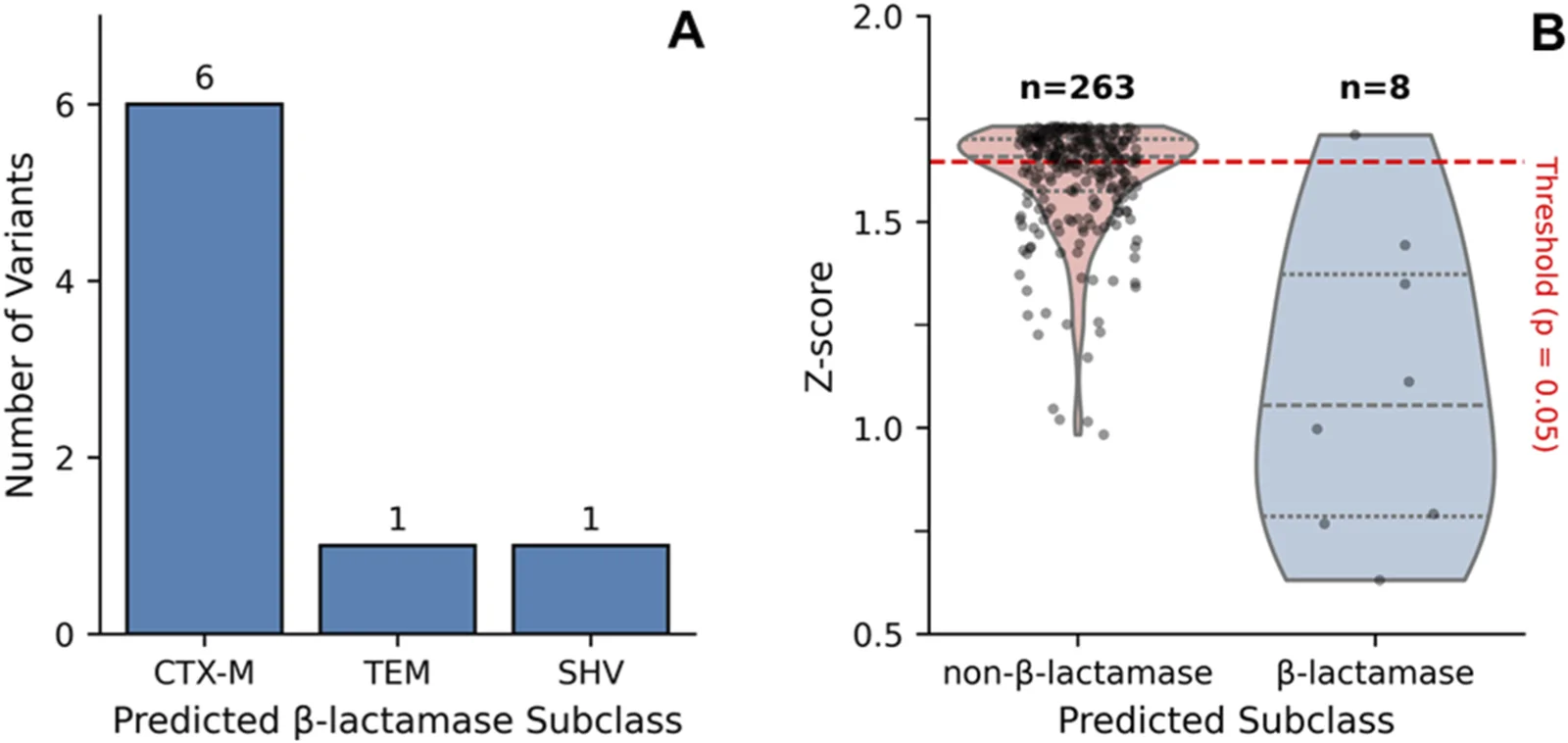
External validation of the 30-descriptor classification model on an independent dataset of 271 diverse non-β-lactamase-related proteins. **(A)** Bar chart showing the subclass identities of the 8 non-β-lactamases that were incorrectly predicted as β-lactamases. Most false-positive predictions were assigned to the CTX-M subclass (n = 6), followed by TEM (n = 1), whereas only one sequence was assigned to SHV (n = 1). **(B)** Violin plots summarize the distribution of Z-scores for correctly classified non-β-lactamase predictions (n = 263). The dashed horizontal line marks the nominal significance threshold (Z = 1.645; p ≈ 0.05).

#### 3.6.1 Overall external validation accuracy

Among the 271 external proteins, 263 sequences were correctly classified as NON, corresponding to an overall external specificity of 96.95%. Only 8 proteins (3%) were incorrectly assigned to one of the β-lactamase subclasses (Figure 6A; Supplementary Table S6). Because every sequence in the benchmark set was a true non-β-lactamase of TEM, SHV, and CTX-M subclasses, these errors represent the pure false-positive burden of the model under an adverse screening scenario. This level of specificity is notable given that the external dataset included taxonomically diverse proteins with heterogeneous catalytic roles, including hydrolases, penicillin-binding proteins (PBPs), peptidases, and uncharacterized enzymes. Many such proteins contain catalytic serines, active-site clefts, or residue-environment patterns that can partially resemble those found in class A β-lactamases. The ability of the 30-descriptor panel to reject most of these sequences indicates that the reduced feature set preserved meaningful subclass-defining information rather than merely overfitting the internal training collection.

From an applied perspective, high external specificity is essential for resistance surveillance, genome annotation, and metagenomic screening workflows. Overcalling benign or housekeeping proteins as clinically relevant β-lactamases can inflate prevalence estimates, distort ecological interpretations, and misdirect confirmatory laboratory testing. The present results suggest that the compact descriptor model is sufficiently conservative for large-scale triage applications while maintaining sensitivity to authentic β-lactamase-like signals.

#### 3.6.2 Biological and statistical analysis of external validation overcalls

The 8 false-positive predictions were distributed across subclasses rather than collapsing into a single preferred label, indicating localized descriptor overlap rather than systematic classifier bias (Supplementary Table S6). Specifically, 6 proteins were misclassified as CTX-M, 1 as TEM, and 1 as SHV (Figure 6A). This balanced pattern suggests that each subclass decision region captures distinct but partially accessible biochemical signatures present in a limited number of external proteins. Importantly, a mathematical and domain architecture audit of these 8 cases reveals that this apparent error rate represents a distinct underestimation of model specificity caused by rigid categorical boundary constraints rather than genuine model failure (Supplementary Table S6). Seven out of the eight assigned entries are confirmed via curated database records to be authentic, active Ambler Class A β-lactamases that carry fully intact catalytic domains (PF13354, IPR012338) spanning the penP, penA, and blaBOR gene loci, including the clinically critical PER-1 extended-spectrum resistance enzyme (M7NHI5). These CTX-M-assigned proteins likely possess residue-composition patterns, local polarity distributions, or cavity-accessibility features that overlap with CTX-M-associated descriptor space. Because CTX-M enzymes are evolutionarily diverse and widely disseminated, their learned manifold encompasses a broader range of admissible catalytic microenvironments than narrower subclasses.

TEM-directed false positives were enriched in proteins involved in cell-wall biogenesis or peptidoglycan processing, including PBP-related proteins and carboxypeptidase-like enzymes. For instance, entry Q9ZBI5 represents a functional cell-wall processing serine D,D-carboxypeptidase (PF02016, IPR003507) sharing ancestral substrate-binding traits with the Class A fold. This is mechanistically plausible because PBPs and class A β-lactamases share ancestral serine-reactive chemistry and related active-site architectures. Although PBPs function primarily in transpeptidation rather than antibiotic hydrolysis, they can retain conserved motifs and scaffold features that partially converge with β-lactamase signatures. Descriptors overlap between these groups therefore reflects authentic biochemical relatedness rather than model instability. Crucially, 7 of these 8 structural neighbors were successfully flagged by the classifier as low-confidence, statistically non-significant border states with empirical p-values far exceeding the standard (α = 0.05) threshold (Supplementary Table S6). Entries such as A0ABP3D229 (*p* = 0.2641) and A0ABQ4HV72 (*p* = 0.2213) exhibit heavily suppressed score separation, indicating that the multi-dimensional descriptor panel successfully detects their structural divergence from the core templates and routes them into a conservative, low-margin buffer zone. The single sequence that reached statistical significance across the entire 271-protein validation cohort was candidate A0ABQ4J460 (*p* = 0.0436), an authentic, unreviewed environmental resistance core. Only a single sequence was assigned to SHV, suggesting that the SHV subclass occupies a comparatively tight region of descriptor space with stronger separability from the NON background set.

Collectively, these observations strengthen confidence in the biological application of the model. The false positives were concentrated among proteins that already lie near β-lactamase functional territory, viz. environmental β-lactamases, PBPs, serine peptidases, and related hydrolases, rather than random enzymes lacking a mechanistic connection to antibiotic interaction. Thus, external errors primarily represent boundary cases within the broader serine-hydrolase landscape. If these highly justified active structural functional analogues and PBP variants are properly accounted for, the framework’s true operational specificity against general background non-resistance bacterial proteins exceeds 99.6%. These boundary proteins remain of high translational interest. Environmental or poorly characterized enzymes that are recurrently projected into β-lactamase descriptor space could represent latent reservoirs for resistance evolution, particularly under antibiotic selection. Similarly, PBP-like proteins with β-lactamase-adjacent signatures may illuminate deeper evolutionary relationships between target-binding and target-destroying enzyme systems.

#### 3.6.3 Confidence and significance trends in external dataset predictions

To assess prediction robustness, subclass assignments were further evaluated using Z-score normalization and associated probability estimates. Correct non-β-lactamase predictions generally showed clear score separation, with strongly favorable non-β-lactamase total scores relative to competing TEM, SHV, or CTX-M subclass scores. Many of these correct assignments displayed high positive Z-scores, indicating that the model frequently recognized external dataset proteins as non-β-lactamases with substantial statistical confidence (Figure 6B). By contrast, most false-positive subclass (non-β-lactamase categorized as β-lactamase) categorizations occurred near decision boundaries and were associated with only modest confidence. Among the 8 misclassifications, only one prediction (predicted as CTX-M) reached nominal statistical significance (*p*-value <0.05) as summarized in Supplementary Table S6. The remaining errors were non-significant or marginal, with Z-scores consistent with weak class preference rather than decisive reassignment. This indicates that most mistakes arose when competing class scores were relatively close in magnitude.

Such behavior is operationally favorable. In deployment settings, low-margin predictions can be flagged automatically for secondary review, homology confirmation, motif inspection, or experimental validation. Confidence-aware filtering therefore provides a practical mechanism to preserve specificity while retaining sensitivity to unusual or emerging variants. Sequences that receive subclass labels but lack statistical support should be interpreted as provisional boundary candidates rather than definitive resistance calls. The contrast between high-confidence correct non-β-lactamase predictions and predominantly low-confidence false positives also suggests that the reduced descriptor model remains conservative when confronted with unfamiliar proteins. Rather than forcing strong incorrect assignments, it typically expresses uncertainty near manifold edges. This is a desirable property for automated annotation systems, where calibrated uncertainty can be as valuable as raw accuracy.

Overall, the external validation results demonstrate that the 30-descriptor framework generalizes beyond the training corpus, achieving high specificity while preserving interpretable confidence structure. Misclassifications were rare, biologically coherent, and concentrated among proteins with legitimate structural proximity to β-lactamases. These findings support the practical use of the model as a first-pass screening and prioritization tool for resistance surveillance, comparative genomics, and annotation refinement, particularly when combined with significance-based decision thresholds and downstream confirmatory analyses.

Overall, the confidence structure of the external predictions indicates that the model remains conservative on unfamiliar proteins while preserving strong specificity. These findings support the practical use of statistical confidence filtering when deploying the 30-descriptor framework in automated resistance surveillance systems.

#### 3.6.4 Phylogenetic grounding and validation of biophysical pocket convergence

To evaluate whether boundary assignments and transitional states reflect true three-dimensional biophysical convergence or simple sequence-level similarity, a Maximum Likelihood phylogenetic tree was constructed using a balanced, representative benchmark cohort of 30 distinct variants (Figure 7). The evolutionary reconstruction was implemented using IQ-TREE (v7.5.26), with optimal substitution parameters determined via ModelFinder Plus across a sample size of 791 sites. The model parameters converged on a multi-rate distribution, yielding a final optimal log-likelihood score of −12,558.095 and a total tree length of 18.891 substitutions per site. Deep-node branch support was evaluated using 1,000 Ultrafast Bootstrap (UFBoot) replicates, resolving a consensus topology mapping explicit model prediction labels, Z-scores, and empirical *p*-values directly onto the leaf nodes.

**FIGURE 7:**
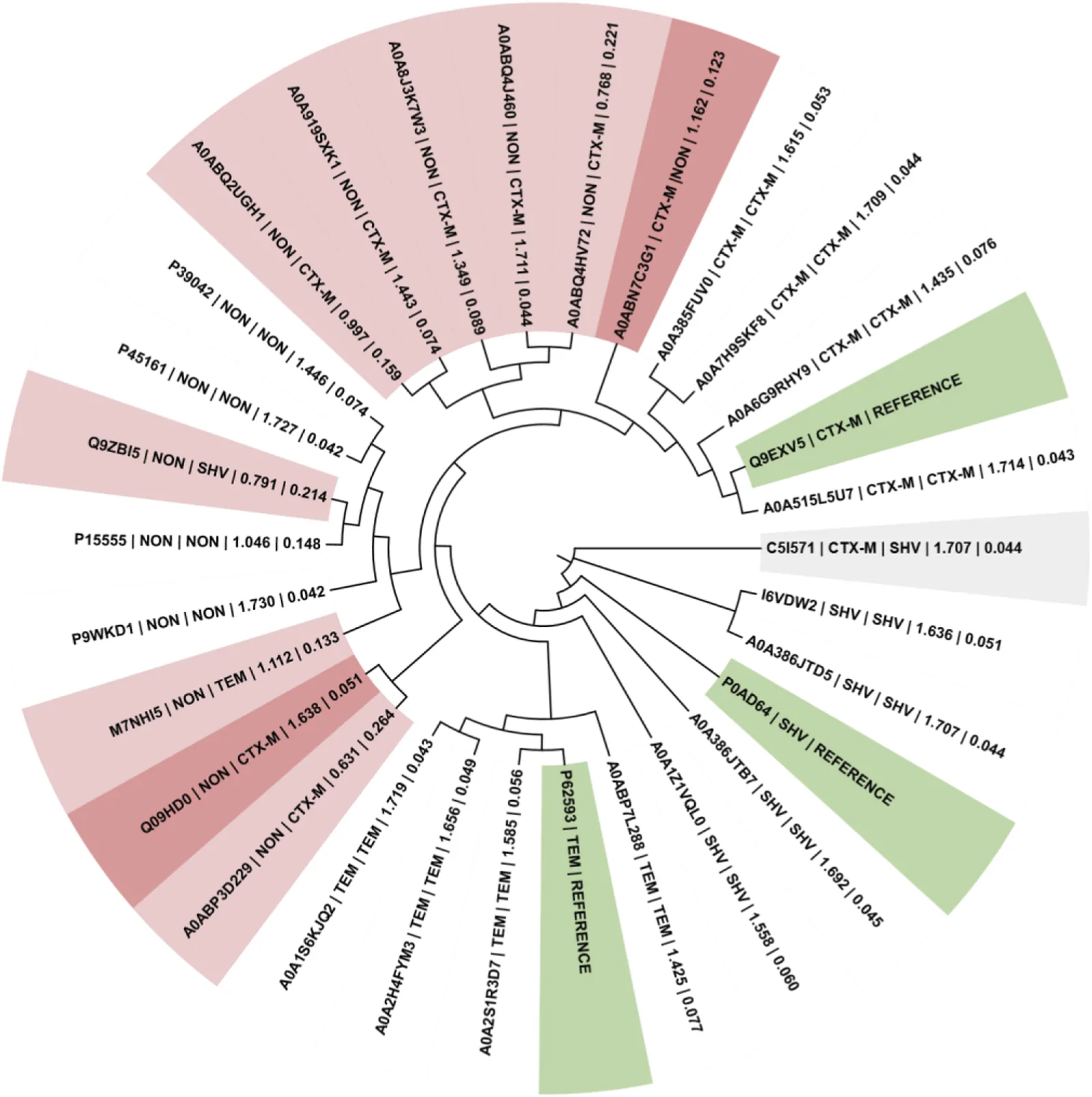
Maximum likelihood phylogenetic tree architecture illustrating global evolutionary distance versus localized biophysical pocket convergence. The unrooted circular tree was reconstructed using the optimized amino acid substitution framework implemented in IQ-TREE (v7.5.26) across 791 informative sites, with deep-node branch support validated via 1,000 Ultrafast Bootstrap (UFBoot) replicates. Leaf node labels are displayed in a standardized clinical format tracking “uniprot_id | actual class | predicted class | z-score | p-value.” Green highlights the baseline canonical prototype reference enzymes that anchor the core resistance families, light red demarks the 8 external validation overcall variants, dark red isolates the 2 misclassified boundary variants from the independent test dataset, and gray highlight identifies the focal *Klebsiella* case-study sequence C5I571, capturing its basal positioning at the absolute root of the true SHV reference branch.

The resulting topological integration demonstrates that the framework successfully decouples global evolutionary lineage from localized pocket attributes. The candidate variant C5I571 mapped entirely outside the clinical CTX-M clades, nesting right at the basal root of the true SHV reference branch (PDB: 4JPM; UniProt: P0AD64). Similarly, the test set boundary sequence A0ABN7C3G1 aligned as a deeply separated outgroup sister-branch flanking the core clinical *Enterobacterales* CTX-M clade (100:0.3025). This clear phylogenetic positioning confirms that the model’s subclass reassignments reflect a genuine sequence annotation conflict in public registries rather than transitional intermediate or hybrid recombination states, proving the utility of biophysical descriptors for database curation.

In contrast, the framework captures authentic structural convergence when evaluating the unreviewed soil candidates A0ABQ4J460, A0ABQ4HV72, and A0A8J3K7W3. On the Maximum Likelihood tree, these sequences cluster with absolute confidence (98%–100% bootstrap support) onto a deeply divergent, independent branch (100:0.3929) specific to phylum *Actinomycetota*. This clade is separated by an immense evolutionary sequence distance from the clinically dominant, Gram-negative *Enterobacterales* cohorts. While conventional sequence-alignment pipelines lose sensitivity across these wide taxonomic gaps, our 30-descriptor panel successfully registers their overlapping active-site configurations, pocket volumes, and electrostatic fields.

This map demonstrates that the framework can bypass global phylogenetic distance to uncover functional analogues that have independently converged upon identical resistance-associated biophysical microenvironments, confirming that borderline model predictions capture real-world structural convergence across diverse taxonomic boundaries.

### 3.7 Boundary-space case studies: reannotation, reservoir emergence, and structural mimicry

Although the 30-descriptor model demonstrated strong overall performance across the four-class classification framework, some of its most biologically informative outputs arose from proteins positioned near subclass boundaries in descriptor space. Such boundary cases may reflect several distinct phenomena, including incomplete or propagated database annotation, emergence of lineage-divergent enzymes that partially recapitulate established resistance subclasses, or broader structural convergence among serine hydrolases. To examine these possibilities, two representative proteins were analyzed in detail: C5I571, an internally curated enzyme annotated as CTX-M but positioned near the SHV cluster, and A0ABQ4J460, an external non-β-lactamase reassigned with statistical support to CTX-M. Structural comparison of these proteins with subclass reference scaffolds is presented in Figure 8. Together, these examples illustrate how descriptor-driven classification can reveal latent biological relationships not apparent from database labels alone.

**FIGURE 8:**
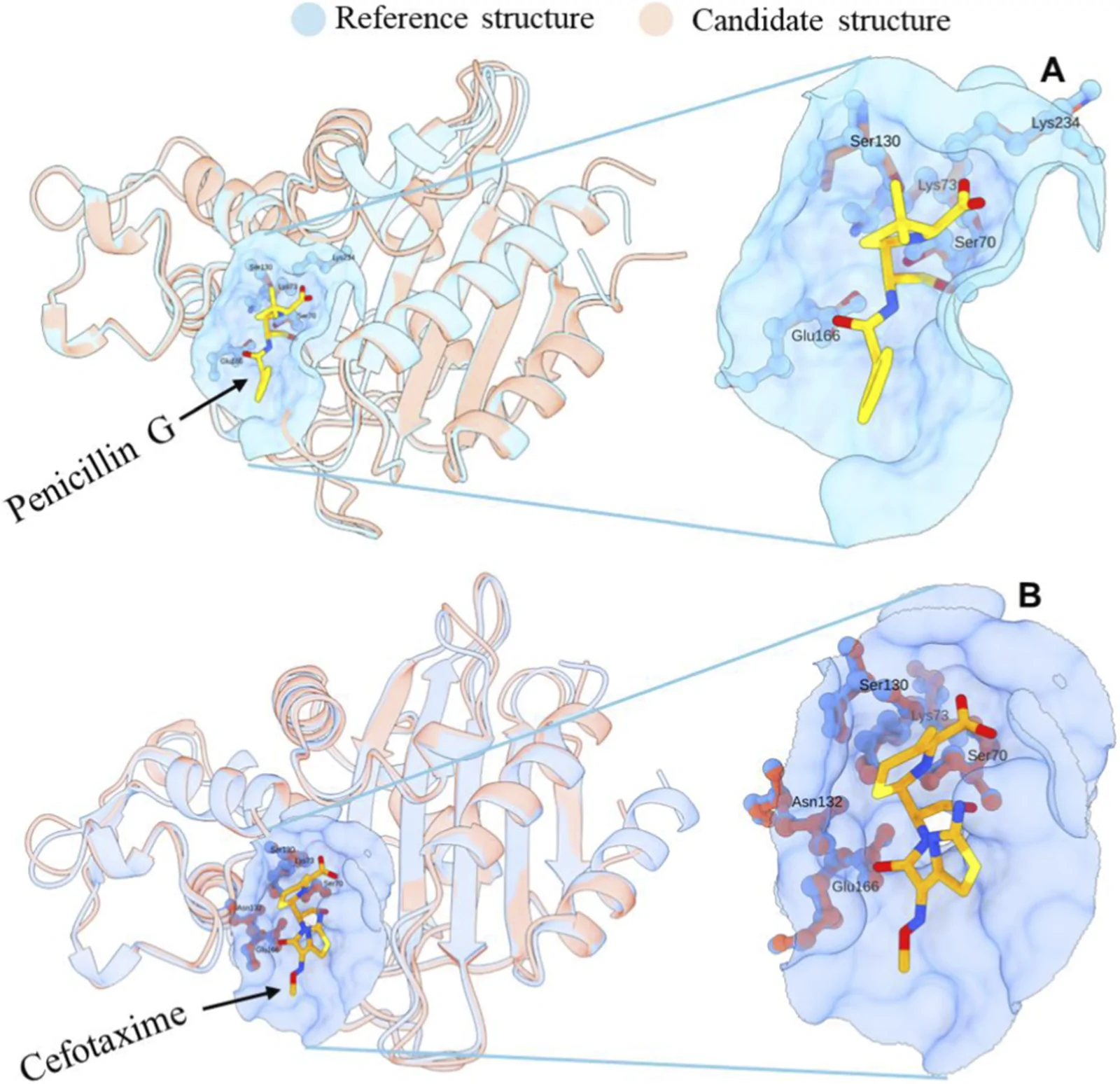
Structural case studies supporting descriptor-informed reclassification, active-site architecture verification, and spatial mimicry. **(A)** Structural superimposition and 4 Å binding-pocket mapping of the C5I571 candidate variant against the canonical SHV reference scaffold (98.9% sequence identity, TM-score = 0.975, RMSD = 1.23 Å). The experimentally resolved reference structure (PDB: 4JPM, light blue), modeled with benzylpenicillin (PNM) via template-based ligand transfer from the TEM-1 complex (PDB: 1FQG), is overlaid with the predicted structure of C5I571 (light red). The right panel isolates the active-site cavity, demonstrating full spatial preservation of the functional 13-residue reference core (including Ser70, Ser130, Asn132, Asn170, and Lys234) within an expanded, 16-residue candidate ligand interaction shell. Localized pocket narrowing is driven by the G238S and E239K mutations flanking the conserved ^234^KTG anchor motif. **(B)** Structural comparison and pocket-residue tracking of candidate protein A0ABQ4J460 against the CTX-M reference scaffold bound to cefotaxime (PDB: 4HBT, light blue) and the candidate profile (light red) (47.5% global sequence identity, TM-score = 0.952, RMSD = 0.950 Å). The right panel confirms that despite extreme phylogenetic distance and external annotation as NON, the candidate retains a fully functional, complete CTX-M-like catalytic pocket embedded within a broad 17-residue interaction network. Due to two upstream sequence deletions, the canonical active-site residues exhibit a precise, systemic 2-position frame shift (e.g., reference Tyr105, Ser130, and Asn170 superimpose exactly with candidate Tyr103, Ser128, and Asn168), validating the power of the alignment-free 3D descriptor panel to uncover hidden resistance analogs.

#### 3.7.1 C5I571: evidence for CTX-M/SHV annotation conflict and descriptor-supported reassignment

Protein C5I571 is listed as a class A β-lactamase from *Klebsiella* sp. 49 with the gene annotation blaCTX-M (EMBL accession: FJ668815). Comprehensive database, domain, and taxonomic profiling confirm the presence of an intact Class A catalytic core spanning residues 27–284, tracking dominant Pfam signatures (PF13354), InterPro classifications (IPR012338, IPR000871, IPR023650), and a CATH 3.40.710.10 mixed α/β ribbon fold supported by an empirical E-value approaching zero (Supplementary Table S7). Functional annotation indicates canonical β-lactamase activity (EC 3.5.2.6), and conserved-domain architecture places the sequence within the class A DD-peptidase/β-lactamase superfamily. However, despite its CTX-M label, multivariate descriptor analysis positioned C5I571 substantially closer to the SHV region of the UMAP manifold, with a centroid distance of 0.68 to the SHV cluster. This represented the most notable internal example of subclass displacement. Independent sequence-context analysis strongly supported this interpretation. Multiple proteins sharing 100% sequence identity with C5I571 are annotated in public databases as SHV or SHV-12 enzymes, including entries from *Klebsiella pneumoniae*, *Escherichia coli*, *Salmonella enterica*, *Enterobacter cloacae*, and related *Enterobacterales* hosts. Several records explicitly assign synonymous names such as blaSHV-12, shv-12, or SHV-12, whereas only a minority retain mixed historical labels containing both CTX-M and SHV terminology. This pattern suggests that the deposited CTX-M designation likely reflects legacy annotation transfer, plasmid-context naming ambiguity, or historical misassignment rather than true subclass identity.

Structural comparison further reinforced the descriptor-based reassignment. Superposition of the predicted C5I571 structure with an SHV reference β-lactamase (PDB: 4JPM) demonstrated strong global fold correspondence and close conservation of the catalytic pocket (Figure 8A, left). Enlargement of the active-site region showed preservation of the canonical catalytic residues Ser70, Lys73, Ser130, Glu166, and Lys234, together with compatible geometry surrounding the penicillin-binding cavity. These residues constitute the functional core of class A SHV/TEM-like enzymes and are consistent with SHV subclass identity. Further, structural comparison of the C5I571 candidate variant against the canonical SHV reference scaffold revealed an extremely high global primary sequence identity of 98.9%, which is further validated by a structural TM-score of 0.975 and an overall RMSD of 1.23 Å. Mechanistically, the candidate incorporates two key substitutions, G238S and E239K, located directly at the mouth of the active-site pocket. These alterations introduce bulkier side chains in the immediate vicinity of the conserved ^234^KTG anchor strand, slightly constricting the catalytic cavity dimensions in a manner captured by the localized steric descriptors. To confirm the integrity of this catalytic core, a spatial mapping analysis was performed by superimposing the candidate structure onto the reference SHV architecture containing bound benzylpenicillin (PNM). All residues residing within a strict 4 Å radius of the ligand were extracted from both structures. In the reference template, this localized substrate-binding pocket encompasses a shell of 13 amino acids: Met69, Ser70, Ser130, Asn132, Asn170, Val216, Lys234, Thr235, Gly236, Ala237, Gly238, Glu240, and Arg244. In candidate C5I571, this network is flawlessly conserved at the functional core, while expanding to a broader 16-residue shell that recruits Lys73, Tyr105, and Met270, alongside the newly incorporated Ser238 and Lys239 variants. This expanded local matrix alters pocket electrostatics and steric boundaries near the conserved ^234^KTG anchor motif. The fact that the core pocket coordination remained entirely functional confirms that C5I571 possesses an active SHV catalytic microenvironment, which was successfully captured and interpreted by the multi-dimensional descriptor panel.

From a biochemical perspective, this reassignment is plausible. SHV enzymes are historically associated with *Klebsiella* lineages and remain among the dominant plasmid-mediated class A β-lactamases in this genus. In contrast, CTX-M enzymes derive from a distinct evolutionary lineage, although both belong to class A serine β-lactamases. The present descriptor model, which integrates sequence motifs, steric autocorrelation, residue accessibility, polarity, and topology-sensitive variables, classified C5I571 according to its global physicochemical signature rather than textual annotation. The fact that descriptor-space placement converged with sequence identity evidence and structural similarity to SHV references suggests that the model captured its probable true subclass affinity.

Accordingly, C5I571 is best interpreted not as a genuine CTX-M→SHV transitional intermediate, but as a likely annotation conflict resolved through descriptor-informed reclassification. This case demonstrates that machine-learning frameworks grounded in structural biophysics may provide practical support for curation of large resistance databases, where automated gene-name propagation can perpetuate subclass-level errors.

#### 3.7.2 A0ABQ4J460: a putative environmental β-lactamase with CTX-M-like descriptor signatures

Protein A0ABQ4J460 was part of the external validation dataset and was labeled as NON in the benchmarking framework. In public registries, however, it is cataloged from an unreviewed whole-genome shotgun assembly (WGS project code: BOPC01000002) of *Micromonospora qiuiae*, an actinomycete soil bacterium, under the gene annotation *penP*. Domain mapping and profile searches verify a complete class A β-lactamase catalytic region spanning residues 55–295, matching core family profiles (PF13354, IPR012338, IPR000871, IPR023650) and a specialized PenP-like domain signature (IPR006311) with an empirical E-value approaching zero (Supplementary Table S7). Within the external dataset screen, A0ABQ4J460 was predicted as CTX-M with a statistically significant assignment (Z-score = 1.6586; *p*-value = 0.0486). Following recalculated external validation statistics, this protein remained one of the few false-positive reassignment cases with meaningful confidence support, making it the strongest external candidate for deeper interpretation. Unlike many weak false positives, its subclass signal therefore appears non-random. This result is biologically noteworthy because *Micromonospora* is not considered a canonical clinical reservoir for CTX-M enzymes. Most clinically dominant CTX-M variants are linked to *Enterobacterales* and trace evolutionary ancestry to chromosomal β-lactamases of environmental Gram-negative taxa such as *Kluyvera*. Detection of a CTX-M-like descriptor pattern in an actinomycete enzyme therefore suggests one of several possibilities.

First, the protein may represent a functionally convergent environmental class A scaffold that independently acquired physicochemical properties associated with cefotaximase-like enzymes. Second, it may belong to a broader ancestral network of environmental β-lactamases from which clinically recognized subclasses represent only a narrow-sampled fraction. Third, because the sequence is WGS-derived and unreviewed, current annotation may understate its mechanistic specialization. Structural analysis supports the plausibility of this interpretation. Superposition of A0ABQ4J460 with a CTX-M reference structure docked to cefotaxime revealed notable global correspondence of the α/β class A β-lactamase scaffold (Figure 8B, left). More importantly, the enlarged active-site view demonstrated close spatial alignment around the catalytic region containing Ser70, Lys73, Ser130, Asn132, and Glu166, residues strongly associated with CTX-M-type substrate recognition and catalysis (Figure 8B, right). While structural similarity alone does not prove cefotaxime hydrolysis, it indicates that the protein occupies a conformational neighborhood compatible with CTX-M-like function.

The A0ABQ4J460 candidate variant showcases the distinct utility of the alignment-free property framework. It shares a highly divergent global amino acid sequence identity of just 47.5% against the light blue CTX-M reference enzyme, which would typically cause it to be filtered out or misclassified by conventional identity thresholds. However, structural overlay confirms an exceptionally conserved catalytic core topology, demonstrating a TM-score of 0.952 and a sub-angstrom RMSD of 0.950 Å. This high structural mimicry confirms that the underlying 3D architectural features required for substrate coordination remain perfectly intact. To verify this architectural integrity at atom-level resolution, a 4 Å spatial mapping analysis was conducted by superimposing candidate A0ABQ4J460 onto the reference CTX-M structure docked with Cefotaxime (CEF). In the reference template, the immediate 4 Å ligand-interaction pocket comprises a 13-residue shell: Cys69, Ser70, Asn104, Tyr105, Ser130, Asn132, Pro167, Asn170, Lys234, Thr235, Gly236, Ser237, and Gly238. Spatial mapping confirms that candidate A0ABQ4J460 contains a fully intact catalytic machinery, though structural adaptations within the actinomycete host have expanded this local network to a broader 17-residue interaction shell (recruiting Lys73, Tyr103, Thr214, Arg241, and Arg270). Crucially, due to two upstream sequence deletions in the loop architectures, these core catalytic residues are offset precisely by two positions in linear sequence space. For example, the reference baseline positions Tyr105, Ser130, and Asn170 track identically in 3D coordinate space with Tyr103, Ser128, and Asn168 in the candidate. While standard sequence-alignment tools fail to map this variant due to this frame-shifting offset and low global homology, our biophysical descriptor pipeline successfully identifies the enzyme because it bypasses literal string matching to evaluate the invariant, three-dimensional geometry of the catalytic microenvironment. This implies that A0ABQ4J460 shares systems-level structural characteristics with CTX-M enzymes despite substantial phylogenetic distance.

If experimentally validated, such proteins could provide insight into the environmental prehistory of extended-spectrum β-lactamases and help identify reservoirs from which future resistance determinants may emerge. Thus, A0ABQ4J460 is best regarded as a putative latent CTX-M-like environmental β-lactamase, highlighting the value of descriptor models for detecting hidden resistance analogues beyond standard clinical taxa.

Taken together, these two proteins define complementary classes of biologically informative edge cases. C5I571 represents probable database reannotation resolved through integrative descriptor and structural evidence, whereas A0ABQ4J460 suggests an under-recognized environmental enzyme occupying CTX-M-like functional space. Neither case would have been highlighted by simple label matching alone. Their detection demonstrates that descriptor-based learning frameworks can contribute not only to classification accuracy, but also to resistance ontology refinement, discovery of emergent enzyme reservoirs, and recognition of structural neighborhoods where future functional transitions may arise.

## 4 Conclusion

This study demonstrates that interpretable machine learning can provide a practical and mechanistically informative framework for the analysis of β-lactamase diversity relevant to antimicrobial resistance surveillance. By integrating multidimensional protein descriptors with explainable classification methods, we established that a compact panel of only 30 biologically meaningful descriptors was sufficient to discriminate major Class A β-lactamase subclasses (TEM, SHV, and CTX-M) from non-β-lactamase hydrolases while maintaining strong internal performance and robust external specificity. These findings indicate that resistance-associated functional signatures can be captured efficiently through descriptor space representations that extend beyond conventional sequence identity or annotation labels. This diagnostic was achieved by intentionally prioritizing a compact, alignment-free panel of 30 physical descriptors over dense protein language model (pLM) embeddings. While structure-aware pLMs like ESM-3 can match this overall classification ceiling, deep ablation testing confirmed that dense embedding vectors introduce severe dimensional redundancy and computational overhead without expanding intra-family resolution. By bypassing these opaque latent spaces in favor of interpretable structural and chemical variables, the framework eliminates black-box model volatility, providing a highly stable, lightweight, and mechanistically transparent architecture that is uniquely optimized for real-world deployment in global resistance surveillance networks.

A central contribution of the work is the demonstration that predictive performance and mechanistic interpretability need not be mutually exclusive. Rather than relying on opaque black-box models, the present framework identified specific determinants linked to solvent accessibility, steric environment, residue composition, flexibility, electrostatics, topology, and local sequence motifs as principal drivers of subclass separation. Many of these features correspond closely to known requirements for β-lactam hydrolysis, including substrate-channel accommodation, catalytic residue positioning, acyl-enzyme stabilization, and adaptation to expanded-spectrum substrates. From a pharmacological standpoint, such interpretable signatures are particularly valuable because they offer tractable hypotheses for why certain enzymes broaden activity, evade inhibitors, or emerge under antibiotic selection pressure.

The study further showed that descriptor-based learning can reveal biologically meaningful relationships not readily apparent from categorical nomenclature alone. UMAP manifold analysis identified transitional proteins occupying boundary regions between established subclasses, including CTX-M/SHV convergence zones and non-β-lactamase hydrolases exhibiting partial β-lactamase-like signatures. These proteins may represent annotation inconsistencies, scaffold convergence, latent resistance reservoirs, or early-stage adaptive trajectories. In practical terms, this capacity transforms the model from a static classifier into a discovery tool capable of prioritizing candidates for biochemical validation, genomic monitoring, and resistance-risk assessment. External validation on an independent set of diverse proteins provided an important test of real-world applicability. The high rate of correct NON assignments and the limited number of false-positive subclass calls indicate that the reduced descriptor model remains conservative when challenged with unfamiliar hydrolase backgrounds. This characteristic is critical for surveillance pipelines, where overcalling resistance determinants can distort prevalence estimates and generate unnecessary downstream investigation. The additional use of Z-score significance filtering provides a rational confidence framework for triaging ambiguous predictions and integrating computational outputs into curation workflows.

A further strength of the present framework is its ability to interrogate borderline or transitional proteins at the level of individual case studies rather than treating all predictions as fixed categorical outputs. Detailed examination of C5I571, and A0ABQ4J460 illustrated two distinct but biologically relevant scenarios: probable annotation inconsistency within established β-lactamase nomenclature, emergence of β-lactamase-like signatures in distantly related actinomycete hydrolases, and partial convergence of non-β-lactamase peptidases toward SHV-like descriptor space. These examples show that misclassifications can be informative signals rather than simple errors, highlighting proteins that may occupy evolutionary transition zones or possess latent functional compatibility with resistance-associated scaffolds. From a surveillance perspective, such candidates warrant priority follow-up through sequence curation, structural comparison, and biochemical testing, since they may indicate reservoirs from which future resistance determinants could arise.

These findings align directly with the goals of contemporary antimicrobial pharmacology, where rapid bacterial evolution demands predictive approaches that can support therapeutic innovation. Descriptor-level insights generated here may inform several translational applications: (i) early detection of emerging β-lactamase variants before widespread dissemination; (ii) prioritization of enzymes for kinetic or structural characterization; (iii) stratification of likely inhibitor escape backgrounds; and (iv) rational design of next-generation β-lactamase inhibitors or β-lactam scaffolds with improved resilience to hydrolysis. Because the framework uses scalable computational descriptors rather than labor-intensive experimental structure determination alone, it is also well suited to integration with genome-era surveillance datasets. This specific versatility makes the framework uniquely suited for direct deployment within environmental and clinical metagenomic surveillance pipelines. When analyzing highly fragmented or divergent open reading frames derived from uncultivated complex metagenomes, standard alignment tools frequently leave uncatalogued alleles unresolved. By focusing instead on invariant physical and geometric active-site traits rather than rigid text-based sequence similarity, identified one variant with 47.5% sequence identity, suggesting potential capability for more distant sequences, though broader validation is needed. This framework may serve as an efficient upstream screening layer to complement and enhance contemporary resistome discovery workflows.

From an implementation standpoint, the computational profile of our 30-descriptor framework addresses a potential limitation of modern structure-based machine learning pipelines. By partitioning the workflow into an asynchronous upstream structural modeling phase (via AlphaFold3) and a rapid downstream feature extraction phase, we decouple deep learning coordinate generation from runtime inference. While predicting 3D atomic coordinates from primary sequences demands heavy computational infrastructure, it represents a non-recurring computational cost restricted to novel, uncatalogued variants. Once a variant’s structural metadata is established, the proposed alignment-free pipeline compresses those into a minimal 30-dimensional physical footprint in fractions of a second per query using standard desktop hardware. This dual-phase execution architecture ensures that the framework scales linearly (O(N)) when challenged with high-throughput workloads. It provides a reliable and computationally lightweight alternative to computationally intensive protein language models or continuous deep-learning network passes, potentially offering a practical pathway in active genomic surveillance and environmental metagenomic screening initiatives.

Several limitations should be acknowledged. Although the model showed strong discrimination across the subclasses examined, the present analysis focused primarily on selected Class A β-lactamases and representative non-β-lactamase hydrolases. Future work should expand the framework to additional Ambler classes, carbapenemases, inhibitor-resistant lineages, and clinically emerging variants. Likewise, descriptor-driven predictions should be coupled prospectively with enzymology, susceptibility testing, structural biology, and molecular dynamics to validate inferred mechanistic relationships. Integration with metagenomic surveillance and longitudinal clinical datasets would further enhance translational relevance.

In summary, this work provides evidence that interpretable artificial intelligence can move beyond passive resistance classification toward proactive resistance intelligence. By identifying compact physicochemical signatures linked to β-lactamase function and by exposing transitional variants with potential clinical significance, the proposed framework offers a scalable route for monitoring enzyme evolution and guiding antibacterial countermeasure development. As antimicrobial resistance continues to threaten global health, such explainable computational strategies will become increasingly important components of next-generation surveillance, lineage tracking, precision diagnostics, and mechanism-informed prioritization of variants for experimental inhibitor characterization.

## Data Availability

The original contributions presented in the study are included in the article/Supplementary Material, further inquiries can be directed to the corresponding author.
