# Supplementary material for: Interpretable machine learning classifies β-lactamase subclasses and prioritizes boundary variants for antimicrobial resistance surveillance
Source: Front Pharmacol. 2026 Sep 25;17:1876559. doi: 10.3389/fphar.2026.1876559 (PMC13649423; doi:10.3389/fphar.2026.1876559)
Supplement: Supplementary file 1 [file Table1.docx]

**SUPPLEMENTARY**

**Table S1.** Species-level taxonomic distribution of the curated non-redundant Class A β-lactamase dataset (n = 274).

| Species / Taxon | TEM | SHV | CTX-M | Total |
| --- | --- | --- | --- | --- |
| *Escherichia coli* | 29 | 4 | 58 | 91 |
| *Klebsiella pneumoniae* | 11 | 54 | 25 | 90 |
| *Salmonella enterica* | 8 | 0 | 8 | 16 |
| *Klebsiella oxytoca* | 0 | 1 | 3 | 4 |
| *Klebsiella aerogenes* | 2 | 1 | 0 | 3 |
| *Klebsiella variicola* | 1 | 0 | 0 | 1 |
| *Kluyvera ascorbata* | 0 | 0 | 3 | 3 |
| *Kluyvera georgiana* | 1 | 0 | 2 | 3 |
| *Kluyvera intermedia* | 0 | 0 | 1 | 1 |
| *Kluyvera genomosp. 2* | 0 | 0 | 1 | 1 |
| *Kluyvera genomosp. 3* | 0 | 0 | 1 | 1 |
| *Kluyvera sichuanensis* | 0 | 0 | 1 | 1 |
| *Enterobacter cloacae* | 1 | 1 | 4 | 6 |
| *Enterobacter hormaechei* | 3 | 0 | 1 | 4 |
| *Enterobacter kobei* | 0 | 0 | 1 | 1 |
| *Enterobacter ludwigii* | 0 | 0 | 1 | 1 |
| *Enterobacter asburiae* | 1 | 0 | 0 | 1 |
| *Citrobacter freundii* | 1 | 0 | 1 | 2 |
| *Proteus mirabilis* | 1 | 0 | 3 | 4 |
| *Proteus vulgaris* | 0 | 0 | 1 | 1 |
| *Providencia stuartii* | 1 | 0 | 1 | 2 |
| *Morganella morganii* | 0 | 0 | 1 | 1 |
| *Serratia marcescens* | 0 | 1 | 1 | 2 |
| *Raoultella terrigena* | 0 | 1 | 0 | 1 |
| *Atlantibacter hermannii* | 0 | 0 | 1 | 1 |
| *Cronobacter malonaticus* | 0 | 1 | 0 | 1 |
| *Shigella sonnei* | 1 | 0 | 0 | 1 |
| *Shigella* sp. | 1 | 0 | 0 | 1 |
| *Xenorhabdus taiwanensis* | 0 | 0 | 1 | 1 |
| *Gibbsiella dentisursi* | 1 | 0 | 0 | 1 |
| Unclassified *Escherichia* spp. | 0 | 0 | 7 | 7 |
| Unclassified *Klebsiella* spp. | 0 | 0 | 1 | 1 |
| Other singleton taxa | 11 | 6 | 4 | 21 |
| Total | 73 | 70 | 131 | 274 |

**Table S2.** Mechanistic and functional classification of the curated hard-negative control dataset (n = 274). This control dataset was systematically compiled from reviewed UniProt entries to serve as an adversarial baseline for model training and specificity optimization. Rather than utilizing distant or superficial sequence negatives that can artificially inflate machine learning metrics, the dataset was explicitly populated with biologically informative structural mimics and functional decoys. These include evolutionary ancestors of resistance enzymes such as penicillin-binding proteins (PBPs) and murein endopeptidases, which share highly conserved catalytic cleft topologies and penicillin-binding geometries with active β-lactamases.

| Functional Category / Structural Family | Representative Enzyme (UniProt IDs) | Catalytic/Structural Mechanism | Sequence Count |
| --- | --- | --- | --- |
| Non-Target Ambler Classes (β-Lactamases) | SME-1 (P52682), GES-5 (Q09HD0), KPC-2 (Q9F663), NMC-A (P52663), OXA-48 (Q6XBH1), VIM-7 (Q840P9) | Serine-active or Zinc-dependent cyclic ring hydrolysis | 24 |
| Cell-Wall Peptidases and Penicillin-Binding Proteins | Zinc DD-peptidase (P00733),  DD-endopeptidase MepA (P0C0T5), DD-carboxypeptidase PBP (P39042) | Penicillin-binding clefts / cell-wall cross-linking | 38 |
| Classical Serine Hydrolases | Trypsin (P00775), Streptogrisin-A (P00776), Lysyl endopeptidase (Q7M135), Protease YdgD (P76176) | Serine-nucleophile catalytic triad (peptide cleavage) | 52 |
| Esterases, Lipases and Thioesterases | PETase (A0A0K8P6T7), Cutinase (G9BY57), BioH (A6TF35), Lipase (Q93MW7), Thioesterase TesA (P9WQD5) | Serine-type carboxylic or fatty-acyl ester cleavage | 65 |
| Lactonases and Deacetylases | AHL-lactonase AiiB (A9CKY2), Fuconolactonase (A0A0H3KNC4), Glyoxalase II GloB (P0AC84), LpxC (O67648) | Hydrolysis of cyclic esters (lactones) or amides | 45 |
| Phosphatases and Nucleases | Phosphatase PtpB (I6WXK4), SuhB (P0ADG4), Endonuclease IV (P0A6C1), Exo III (P09030) | Phosphate ester or nucleic acid phosphodiester cleavage | 50 |
| Total Negative Control Dataset |  |  | 274 |

**Table S3.** Taxonomic diversity of the external validation dataset (n = 271 proteins). The external validation dataset spans a broad phylogenetic range of bacterial taxa to minimize lineage-specific bias and ensure that predictive performance was not driven by narrow organismal composition.

| Taxonomic Group | Representative Genera / Organisms in Dataset | Count* |
| --- | --- | --- |
| Proteobacteria | *Escherichia, Pseudomonas, Vibrio, Klebsiella, Salmonella, Acinetobacter, Burkholderia, Caulobacter, Legionella, Morganella* | 96 |
| Firmicutes / Bacillota | *Bacillus, Bacillus-related genera, Clostridium, Enterococcus, Lactobacillus-group taxa, Geobacillus* | 54 |
| Actinobacteria / Actinomycetota | *Mycobacterium, Mycolicibacterium, Streptomyces, Amycolatopsis, Micromonospora, Rhodococcus* | 47 |
| Bacteroidota | *Bacteroides, Phocaeicola, Chryseobacterium, Maribacter, Tenacibaculum, Zobellia* | 22 |
| Cyanobacteria | *Gloeobacter, Planktothrix, Phormidium, Synechocystis, Plectonema* | 11 |
| Mollicutes / Mycoplasma lineage | *Mycoplasma, Mycoplasmoides, Mycoplasmopsis* | 9 |
| Spirochaetota | *Leptospira, Treponema* | 4 |
| Thermophilic deep-branching bacteria | *Thermotoga, Persephonella, Symbiobacterium, Halothermothrix* | 8 |
| Other environmental / less represented lineages | Multiple marine, soil, symbiotic and uncultured taxa | 20 |
| Total |  | 271 |
| *Counts are approximate and collapsed higher-rank taxonomy was used for readability. | | |

**Table S4.** Deep ablation and performance profiling of pre-trained protein language model (pLM) embeddings on the independent validation dataset (n = 164). Performance metrics are reported across sequence-derived (ProtT5), structure-aware multimodal (ESM-3), and concatenated (Combined) embedding spaces, evaluated using both full-dimensional raw feature matrices ("Original") and compressed dimensions capturing exactly 90% of the feature variance ("PCA"). Both macro- and weighted-averaged metrics are provided across the TEM, SHV, CTX-M, and NON lineages. The data demonstrates a critical mathematical ceiling where the concatenated framework introduces computational overhead and feature redundancy without exceeding the predictive accuracy (0.988) or Matthews Correlation Coefficient (0.981) achieved by ESM-3 alone, structurally justifying the model's pivot to an interpretable 30-descriptor classical biophysical framework.

| Language Model | Features | Average Type | Acc. | Sens. | Spec. | Prec. | F1 | MCC |
| --- | --- | --- | --- | --- | --- | --- | --- | --- |
| ProtT5 | Original Features | Macro  Weighted | 0.939 0.939 | 0.935  0.939 | 0.972  0.978 | 0.938  0.950 | 0.932  0.940 | 0.909  0.916 |
|  | PCA  (90% Var) | Macro  Weighted | 91.46  91.46 | 91.16  91.46 | 0.957  0.955 | 0.918  0.923 | 0.911  0.916 | 0.869  0.872 |
| ESM-3 | Original Features | Macro  Weighted | 0.988  0.988 | 0.991  0.988 | 0.994  0.996 | 0.985  0.988 | 0.988  0.988 | 0.981  0.980 |
|  | PCA  (90% Var) | Macro  Weighted | 0.988  0.988 | 0.985  0.988 | 0.992  0.989 | 0.992  0.988 | 0.988  0.988 | 0.981  0.980 |
| Combined | Original Features | Macro  Weighted | 0.976  0.976 | 0.976  0.976 | 0.989  0.991 | 0.970  0.976 | 0.972  0.976 | 0.962 0.964 |
|  | PCA  (90% Var) | Macro  Weighted | 0.988  0.988 | 0.991  0.988 | 0.994  0.996 | 0.985  0.988 | 0.988  0.988 | 0.981  0.980 |
| Acc. – Accuracy; Sens. – Sensitivity; Spec. – Specificity; Prec. – Precision | | | | | | | | |

| **Table S5.** A summary of benchmarking performance comparison against standard sequence-based ARG annotation platforms on the test dataset. Performance metrics were rigorously benchmarked using an independent test dataset (n = 164) across two hierarchical granularities: strict multi-class subclass lineage discrimination (Part A) and macro-level binary family-diagnostic capacity (Part B).While state-of-the-art homology-driven, profile Hidden Markov Model (HMM) pipelines, and protein language models (such as AMRFinder+, CARD-RGI, DeepARG, and PLM-ARG) display robust baseline predictive capacities on canonical reference sequences, their architectures remain inherently constrained by a rigid dependency on predefined, high-identity alignment databases. Consequently, sequence-level variations, localized point mutations, or evolutionary boundary transitions frequently force these platforms to drop back to non-specific family designations (e.g., generic BETA assignments), compromising subclass-specific sensitivity and introducing false-positive drift when exposed to background environmental hydrolases.Method | Accuracy | Sensitivity | Specificity | Precision | F-Score | MCC |
| --- | --- | --- | --- | --- | --- | --- |
| **Part A:** Strict Subclass-Specific Level (TEM vs. SHV vs. CTX-M vs. NON) | | | | | | |
| AMRFinder+ | 0.933 | 0.927 | 0.939 | 0.938 | 0.932 | 0.906 |
| CARD (RGI) | 0.927 | 0.902 | 0.951 | 0.949 | 0.925 | 0.890 |
| DeepARG | 0.945 | 0.915 | 0.976 | 0.974 | 0.943 | 0.921 |
| PLM-ARG | N/A | N/A | N/A | N/A | N/A | N/A |
| Current Study | 0.988 | 0.990 | 0.984 | 0.990 | 0.990 | 0.983 |
| **Part B:** Binary family-diagnostic level (active beta-lactamase vs. control) | | | | | | |
| AMRFinder+ | 0.969 | 1.000 | 0.939 | 0.942 | 0.970 | 0.941 |
| CARD (RGI) | 0.927 | 0.902 | 0951 | 0.949 | 0.925 | 0.855 |
| DeepARG | 0.982 | 0.988 | 0.976 | 0.976 | 0.982 | 0.963 |
| PLM-ARG | 0.969 | 1.000 | 0.919 | 0.953 | 0.976 | 0.936 |
| Current Study | 0.988 | 0.990 | 0.984 | 0.990 | 0.990 | 0.983 |
| *Note: Part A explicitly penalizes generic outputs on target variants as subclass sorting failures, while treating broad BETA tags on true NON sequences as real False Positives to guarantee absolute biological transparency. | | | | | | |

**Table S6.** Misclassified sequences in the external validation dataset with statistical confidence metrics. Statistical assessment of the 8 external validation sequences that were annotated as NON but predicted by the 30-descriptor multiclass model as canonical β-lactamase subclasses (CTX-M, TEM, or SHV). Z-scores were calculated relative to the empirical score distribution generated during model validation and corresponding two-tailed P-values were used to estimate assignment confidence. Only one sequence, A0ABQ4J460 (predicted CTX-M), reached nominal statistical significance (*p-value* < 0.05), indicating that most false-positive calls represented weak or borderline subclass signals rather than robust misclassification. These two statistically supported cases were therefore selected for detailed structural and annotation-based analysis in Section 3.9.

| UniProt ID | Pfam Domain | Organism origin | Gene Name | Actual  Class | Predicted Class | *Z-score* | *p-value* |
| --- | --- | --- | --- | --- | --- | --- | --- |
| A0A8J3K7W3 | PF13354 | *Catellatospora chokoriensis* | penP | NON | CTX-M | 1.272 | 0.102 |
| A0A919SXK1 | PF13354 | *Winogradskya consettensis* | penA | NON | CTX-M | 1.412 | 0.079 |
| A0ABP3D229 | PF13354 | *Methylophaga marina* | blaBOR | NON | CTX-M | 0.630 | 0.264 |
| A0ABQ2UGH1 | PF13354 | *Lentzea flava* | penA | NON | CTX-M | 1.104 | 0.135 |
| **A0ABQ4J460** | PF13354 | *Micromonospora qiuiae* | penP | NON | CTX-M | 1.659 | **0.049** |
| A0ABQ4HV72 | PF13354 | *Micromonospora andamanensis* | penP | NON | CTX-M | 0.768 | 0.221 |
| M7NHI5 | PF13354 | *Bhargavaea cecembensis* | per1 | NON | TEM | 1.112 | 0.133 |
| Q9ZBI5 | PF13354 | *Streptomyces coelicolor* | SCO6489 | NON | SHV | 0.791 | 0.214 |

**Table S7.** Empirical Domain, Curation, and Taxonomic Baseline Verification for Focal Case Study Boundary Variants, C5I571 and A0ABQ4J460.

| Parameter Metric | Case Study 1  Variant: C5I571 | Case Study 2  Variant: A0ABQ4J460 |
| --- | --- | --- |
| UniProt ID | C5I571 | A0ABQ4J460 |
| Organism Origin | *Klebsiella* sp. 49 | *Micromonospora qiuiae* |
| Taxonomic Lineage | Gammaproteobacteria, Enterobacterales | Actinomycetota, Micromonosporales |
| Gene Name | blaCTX-M | penP |
| Assigned Label | Target Dataset (Legacy: CTX-M) | External Validation Dataset (NON) |
| Predicted Class | SHV (Subclass Reassignment) | CTX-M (Borderline Priority Case) |
| Model Z-Score | 0.6800 (Centroid Proximity to SHV) | 1.6586 (High-Margin Signal) |
| Empirical P-Value | Low-Margin Boundary Displacement | 0.0486 (Statistically Significant) |
| Pfam Domain | PF13354  (Beta-lactamase class A catalytic) | PF13354  (Beta-lactamase class A catalytic) |
| InterPro  Architectures | IPR012338 (Beta-lactamase, class A) IPR000871 (Beta-lactamase_A) IPR023650 (Beta-lactamase, Class-A) | IPR012338 (Beta-lactamase, class A) IPR000871 (Beta-lactamase_A) IPR006311 (PenP-like domain) |
| NCBIfam Reference Profile | NF033103  (Class A β-lactamase family) | NF033103  (Class A β-lactamase family) |
| CATH Structural Fold | 3.40.710.10  (Alpha-Beta Ribbon Scaffold) | 3.40.710.10  (Alpha-Beta Ribbon Scaffold) |

**Figure S1.** A workflow of the training and validation of the 30-descriptor based framework. The initial dataset of 548 sequences was partitioned at a ratio of 70:30 for training and independent testing. A set of 30 descriptors was identified entirely from the training dataset to develop a multiclass logistic regression model. This model was evaluated on the independent test dataset and an external dataset of 271 proteins.
